# Supplementary material for: A plasma membrane-localized polycystin-1/polycystin-2 complex in endothelial cells elicits vasodilation
Source: eLife. 2022 Mar 1;11:e74765. doi: 10.7554/eLife.74765 (PMC8933003; doi:10.7554/eLife.74765)
Supplement: Source data 1. [file elife-74765-data1.zip › 100355_1_supp_data_2358340_r6ybjl/Source data legends.docx]

Figure 1-Source Data 1: Original uncropped blot illustrating PC-1 protein in mesenteric arteries of *Pkd1^fl/fl^* and *Pkd1* ecKO mice.

Figure 1-Source Data 2: Original uncropped blot illustrating PC-2 protein in mesenteric arteries of *Pkd1^fl/fl^* and *Pkd1* ecKO mice.

Figure 1-Source Data 3: Original uncropped blot illustrating SK3 protein in mesenteric arteries of *Pkd1^fl/fl^* and *Pkd1* ecKO mice.

Figure 1-Source Data 4: Original uncropped blot illustrating IK protein in mesenteric arteries of *Pkd1^fl/fl^* and *Pkd1* ecKO mice.

Figure 1-Source Data 5: Original uncropped blot illustrating TRPV4 protein in mesenteric arteries of *Pkd1^fl/fl^* and *Pkd1* ecKO mice.

Figure 1-Source Data 6: Original uncropped blot illustrating Piezo1 protein in mesenteric arteries of *Pkd1^fl/fl^* and *Pkd1* ecKO mice.

Figure 1-Source Data 7: Original uncropped blot illustrating eNOS protein in mesenteric arteries of *Pkd1^fl/fl^* and *Pkd1* ecKO mice.

Figure 1 – Source Data 8: Original uncropped blot illustrating GPR68 protein in mesenteric arteries of *Pkd1^fl/fl^* and *Pkd1* ecKO mice.

Figure 1-Source Data 9: Original uncropped blot illustrating actin protein in mesenteric arteries of *Pkd1^fl/fl^* and *Pkd1* ecKO mice.

Figure 1-Figure supplement 1-Source Data 1: Original uncropped genomic PCR gel indicating that tamoxifen stimulated Cre-recombination in mesenteric arteries of *Pkd1^fl/fl^: Cdh5*(PAC)-creERT2 mice.

Figure 2-Source Data 1: Original uncropped blots illustrating p-eNOS protein in mesenteric arteries of *Pkd1^fl/fl^* and *Pkd1* ecKO mice.

Figure 2-Source Data 2: Original uncropped blots illustrating eNOS protein in mesenteric arteries of *Pkd1^fl/fl^* and *Pkd1* ecKO mice.

Figure 2-Source Data 3: Original uncropped blots illustrating actin protein in mesenteric arteries of *Pkd1^fl/fl^* and *Pkd1* ecKO mice.

Figure 4-Source data 1: Original uncropped blots illustrating the detection (IB) of both PC-1 and PC-2 (IB) in PC-2 immunoprecipitate (IP) in mesenteric arteries of *Pkd1^fl/fl^* mice.

Figure 6-Source Data 1: Original uncropped blot illustrating PC-1 protein in mesenteric arteries of *Pkd1^fl/fl^ /Pkd2^fl/fl^* and *Pkd1/Pkd2* ecKO mice.

Figure 6-Source Data 2: Original uncropped blot illustrating PC-2 protein in mesenteric arteries of *Pkd1^fl/fl^ /Pkd2^fl/fl^* and *Pkd1/Pkd2* ecKO mice.

Figure 6-Source Data 3: Original uncropped blot illustrating eNOS protein in mesenteric arteries of *Pkd1^fl/fl^ /Pkd2^fl/fl^* and *Pkd1/Pkd2* ecKO mice.

Figure 6-Source Data 4: Original uncropped blot illustrating actin protein in mesenteric arteries of *Pkd1^fl/fl^ /Pkd2^fl/fl^* and *Pkd1/Pkd2* ecKO mice.
