# Supplementary figures and images for: A plasma membrane-localized polycystin-1/polycystin-2 complex in endothelial cells elicits vasodilation
Source: eLife. 2022 Mar 1;11:e74765. doi: 10.7554/eLife.74765 (PMC8933003; doi:10.7554/eLife.74765)

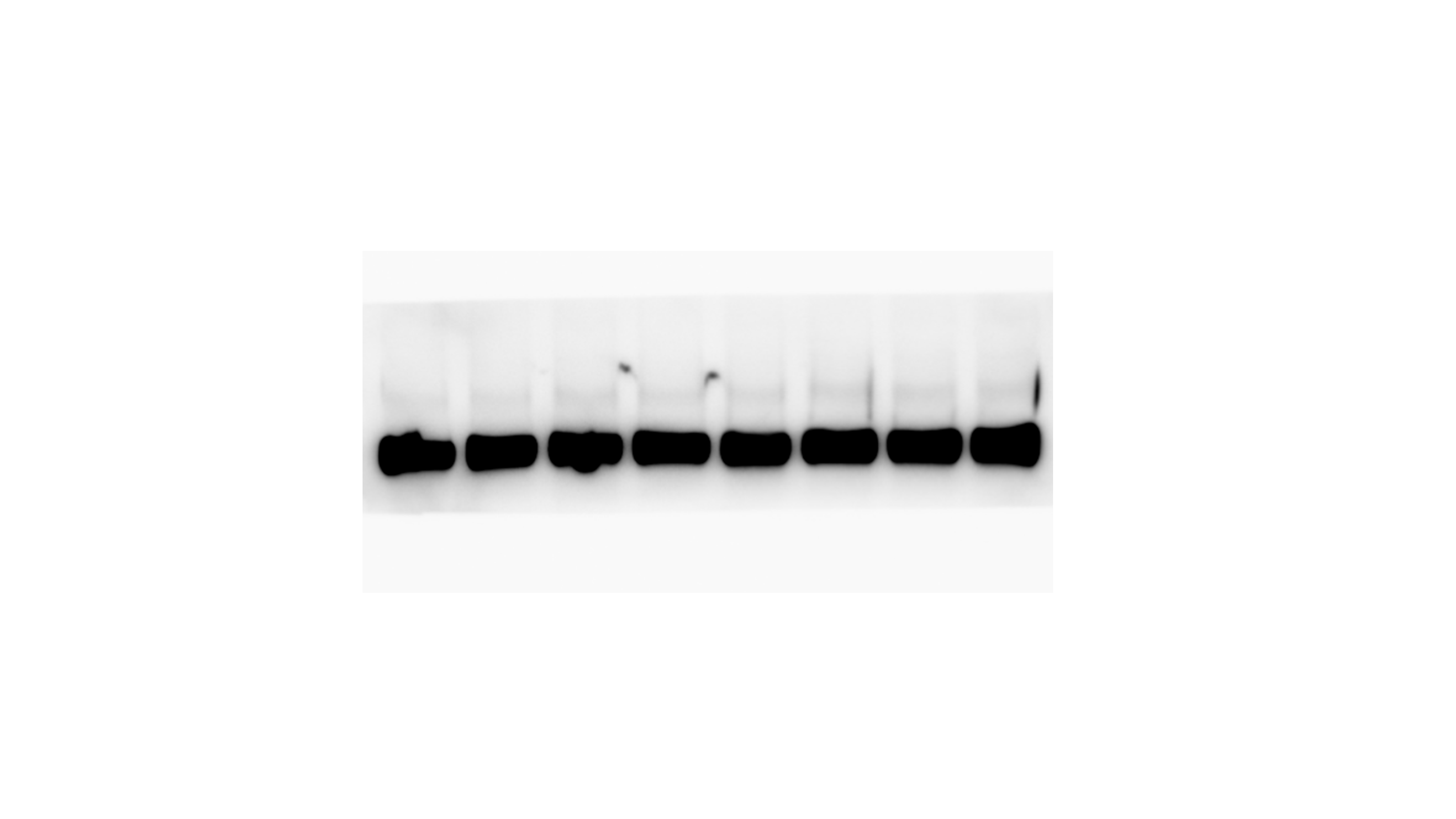

Supplement: Source data 1. [file elife-74765-data1.zip › 100355_1_supp_data_2358340_r6ybjl/Figure 1 - source data 9.TIF]

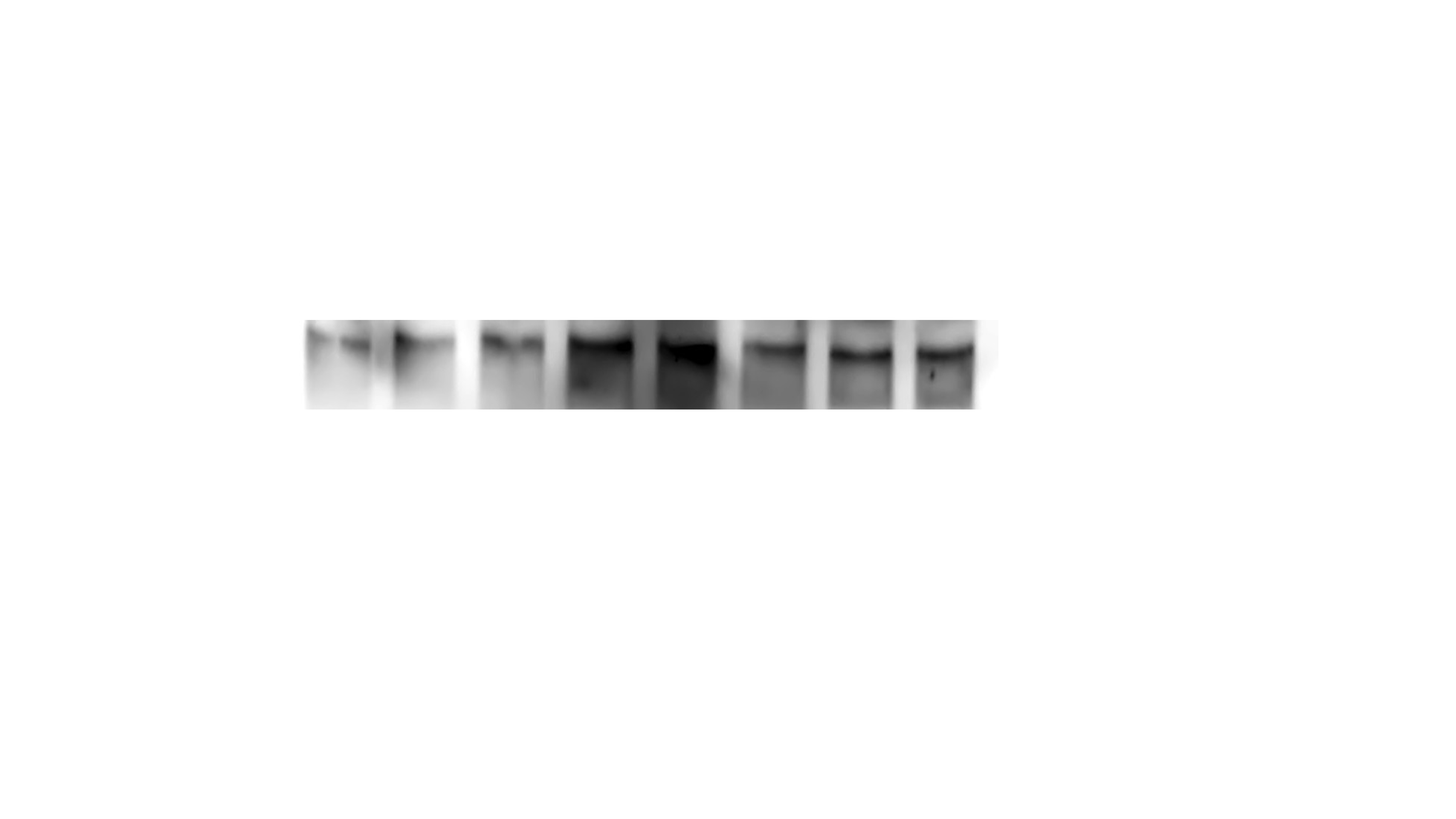

Supplement: Source data 1. [file elife-74765-data1.zip › 100355_1_supp_data_2358340_r6ybjl/Figure 1 - source data 8.TIF]

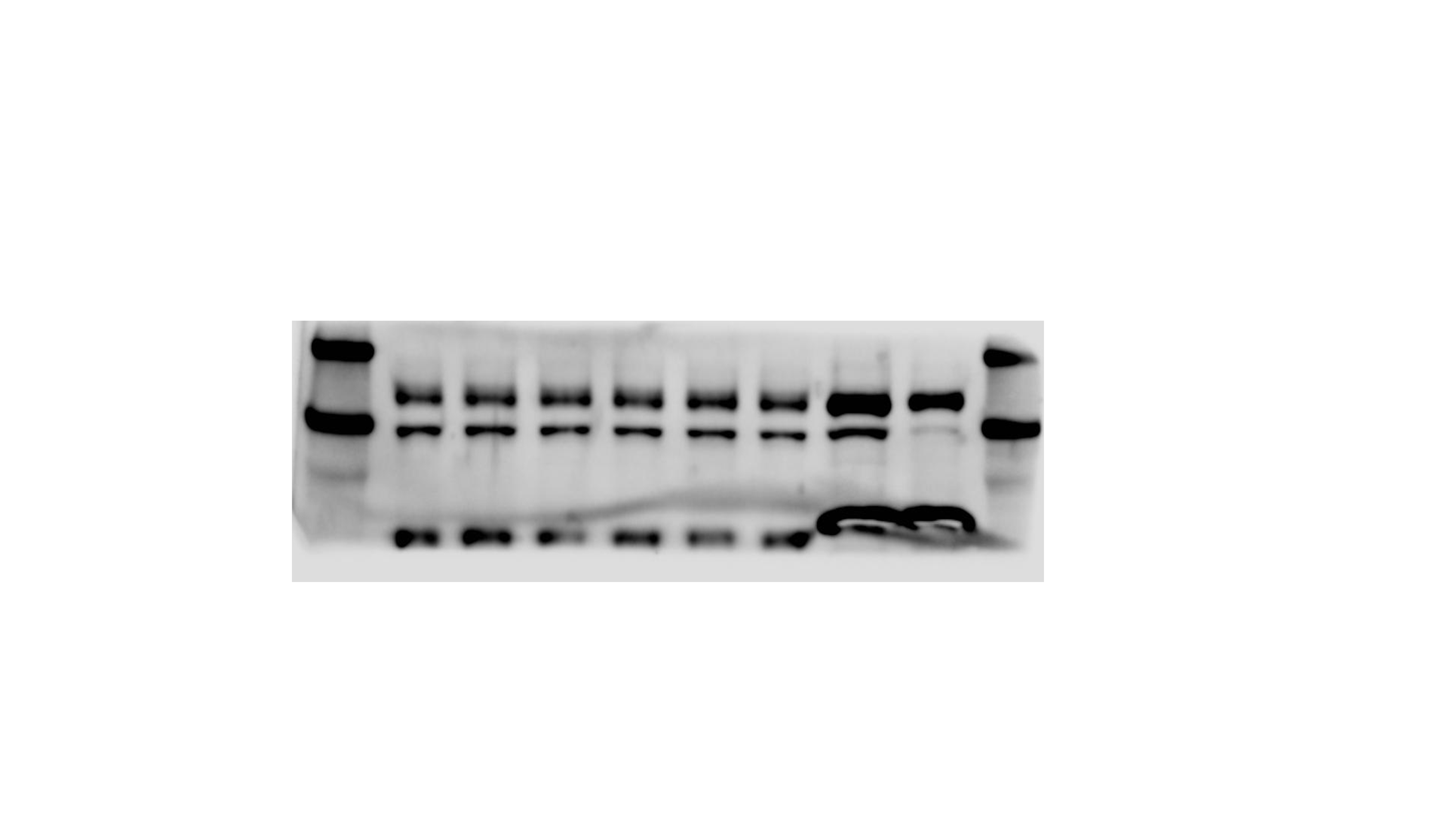

Supplement: Source data 1. [file elife-74765-data1.zip › 100355_1_supp_data_2358340_r6ybjl/Figure 1 - source data 5.TIF]

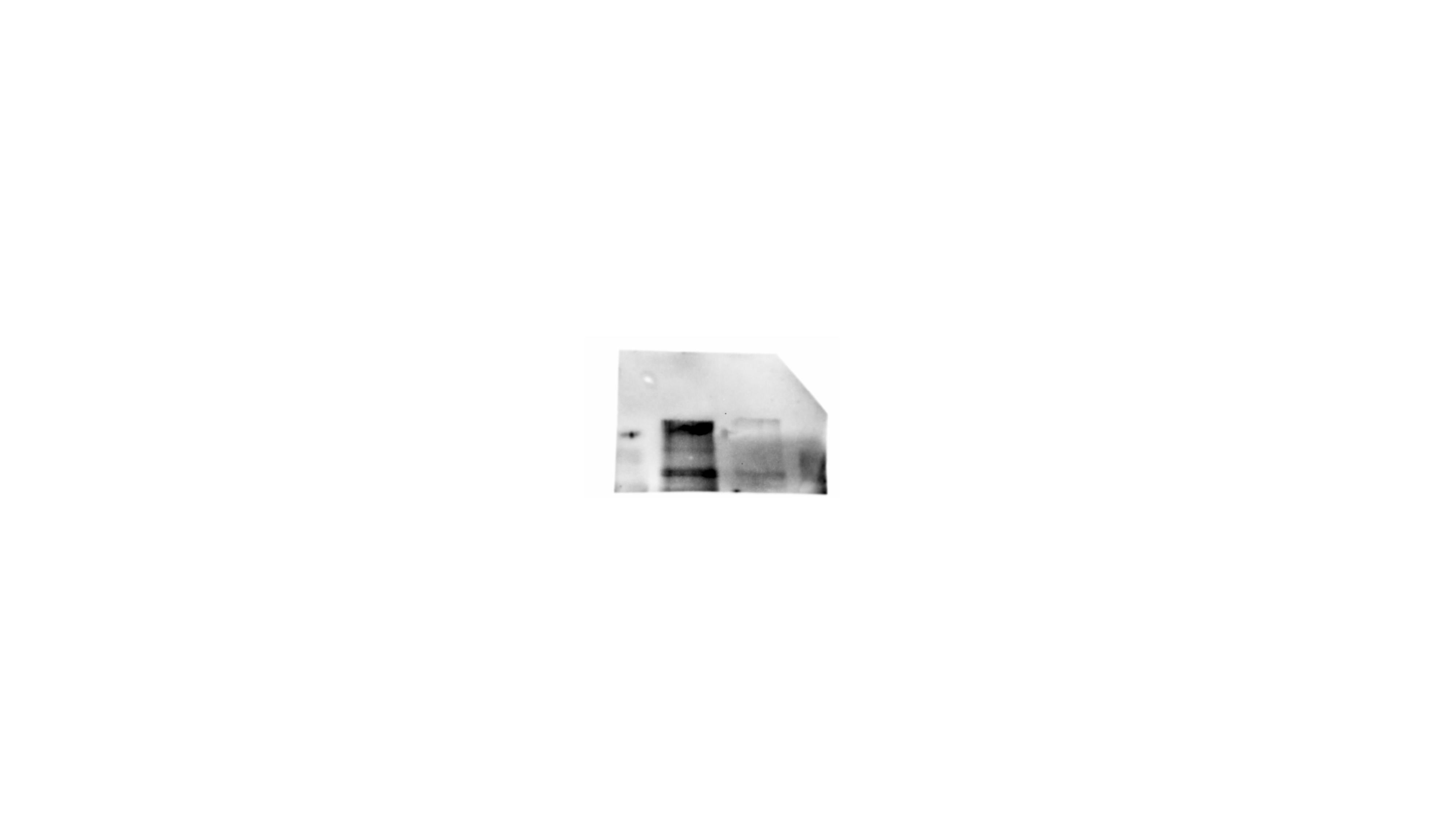

Supplement: Source data 1. [file elife-74765-data1.zip › 100355_1_supp_data_2358340_r6ybjl/Figure 6 - source data 1.TIF]

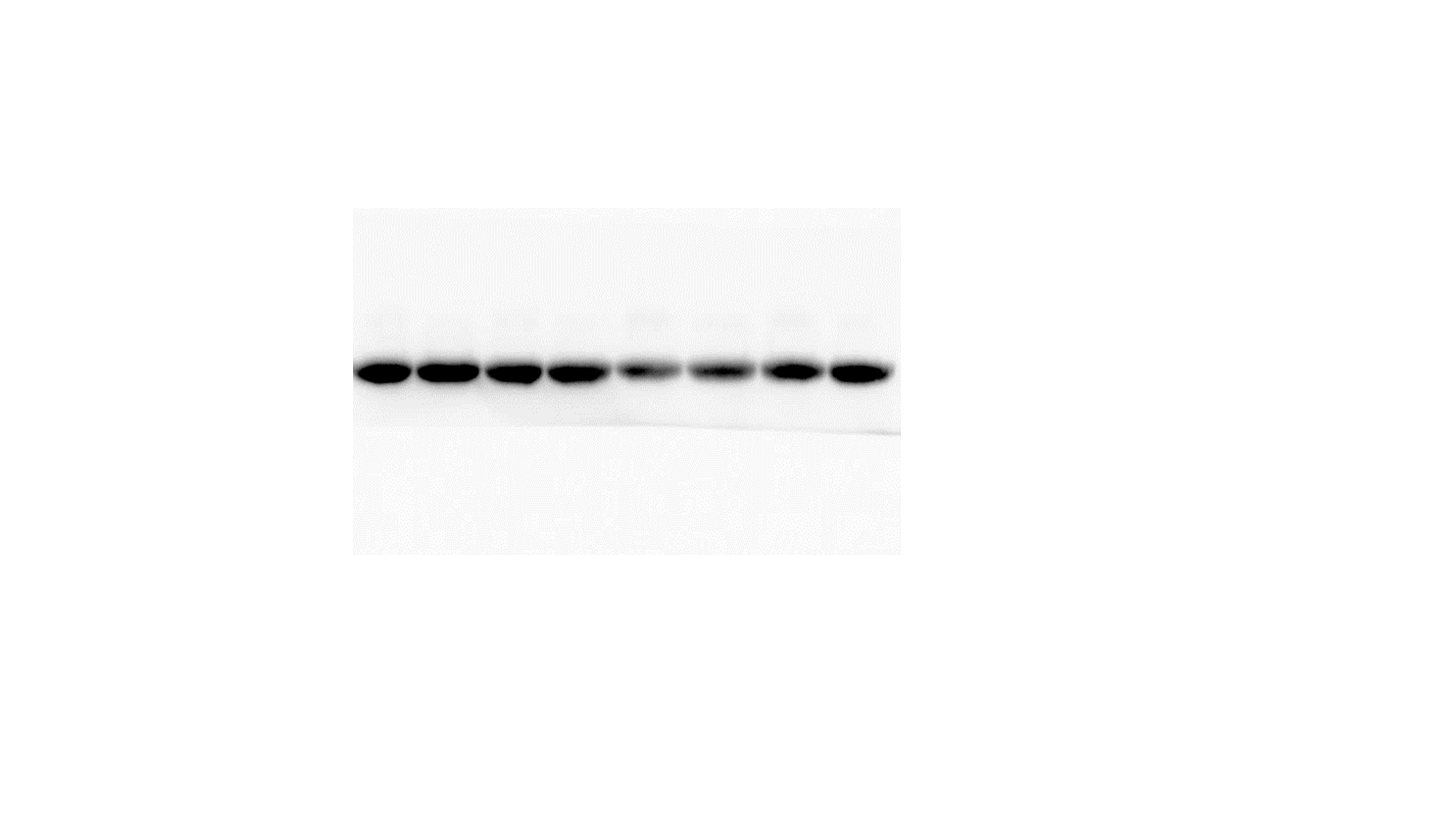

Supplement: Source data 1. [file elife-74765-data1.zip › 100355_1_supp_data_2358340_r6ybjl/Figure 1 - source data 4.TIF]

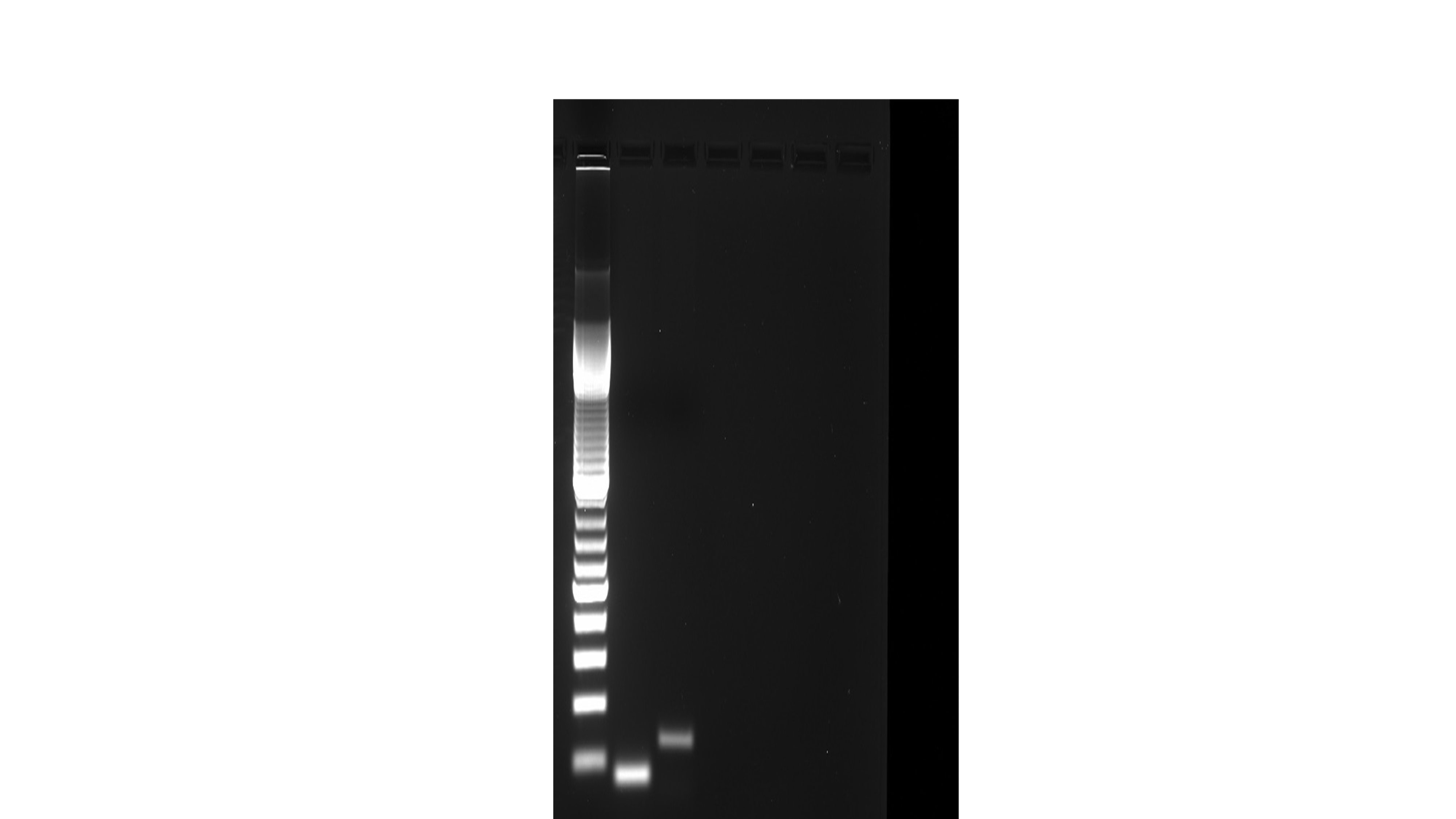

Supplement: Source data 1. [file elife-74765-data1.zip › 100355_1_supp_data_2358340_r6ybjl/Figure 1-Figure Supplement 1-source data 1.TIF]

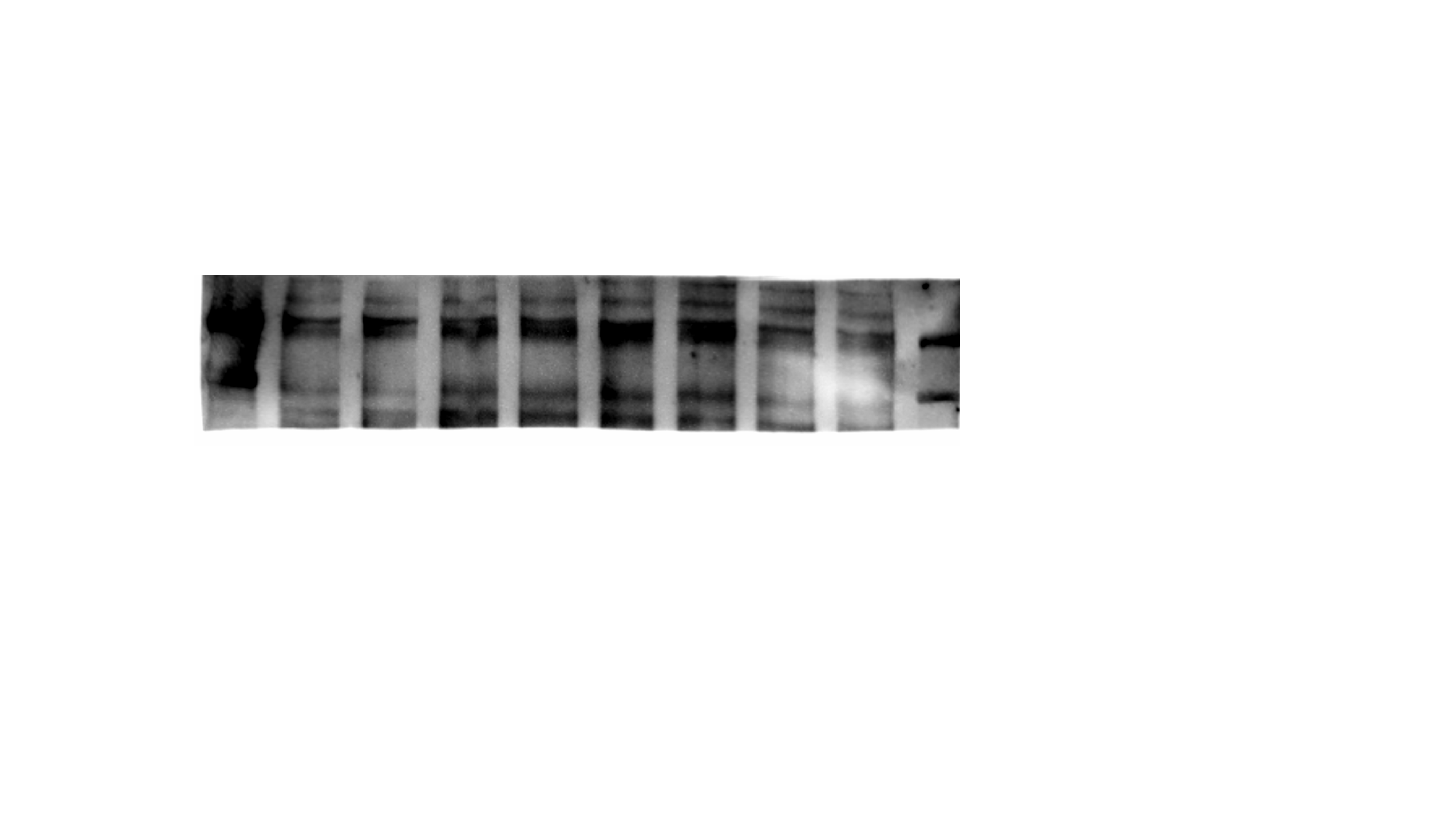

Supplement: Source data 1. [file elife-74765-data1.zip › 100355_1_supp_data_2358340_r6ybjl/Figure 1 - source data 6.TIF]

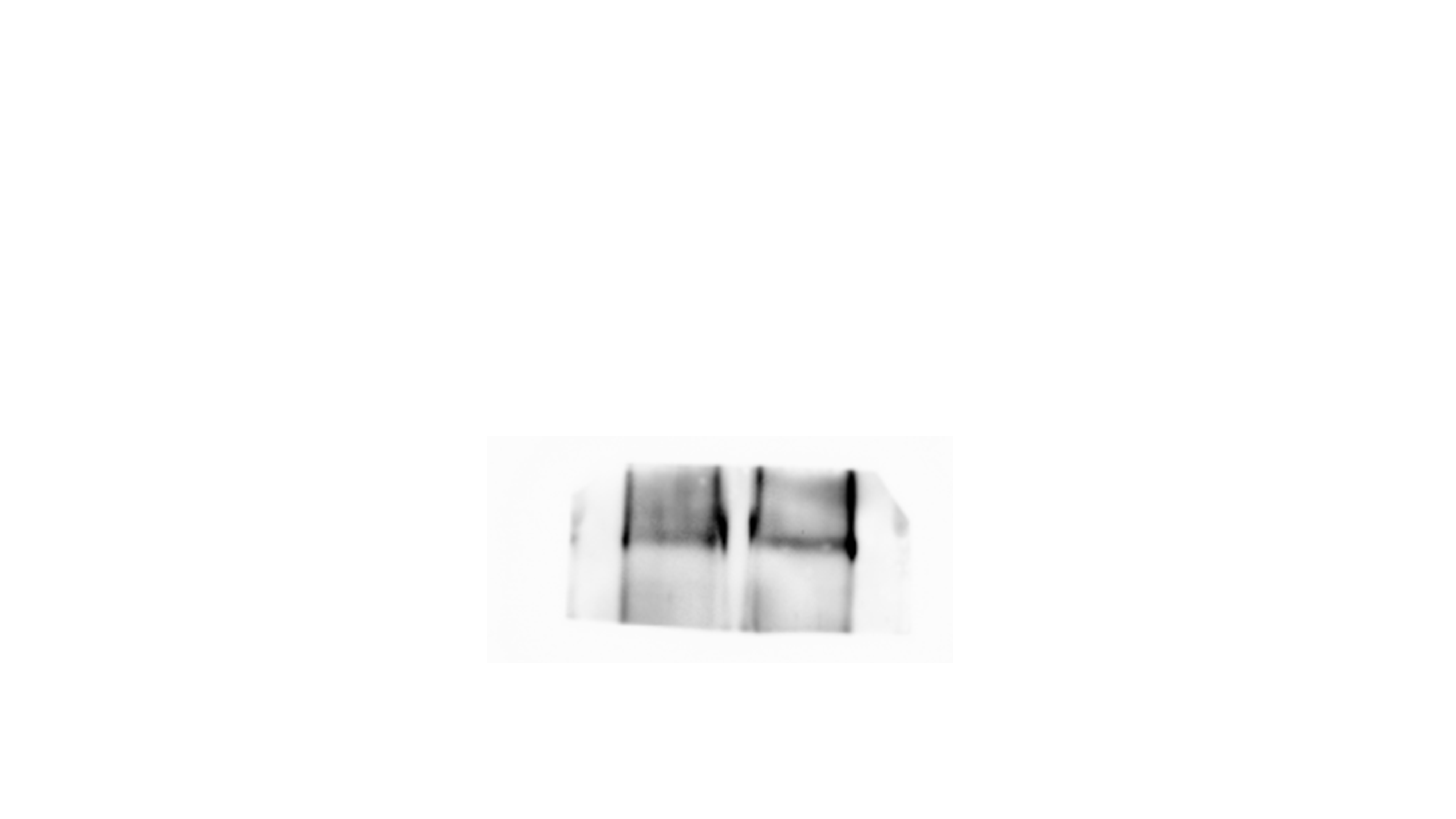

Supplement: Source data 1. [file elife-74765-data1.zip › 100355_1_supp_data_2358340_r6ybjl/Figure 6 - source data 3.TIF]

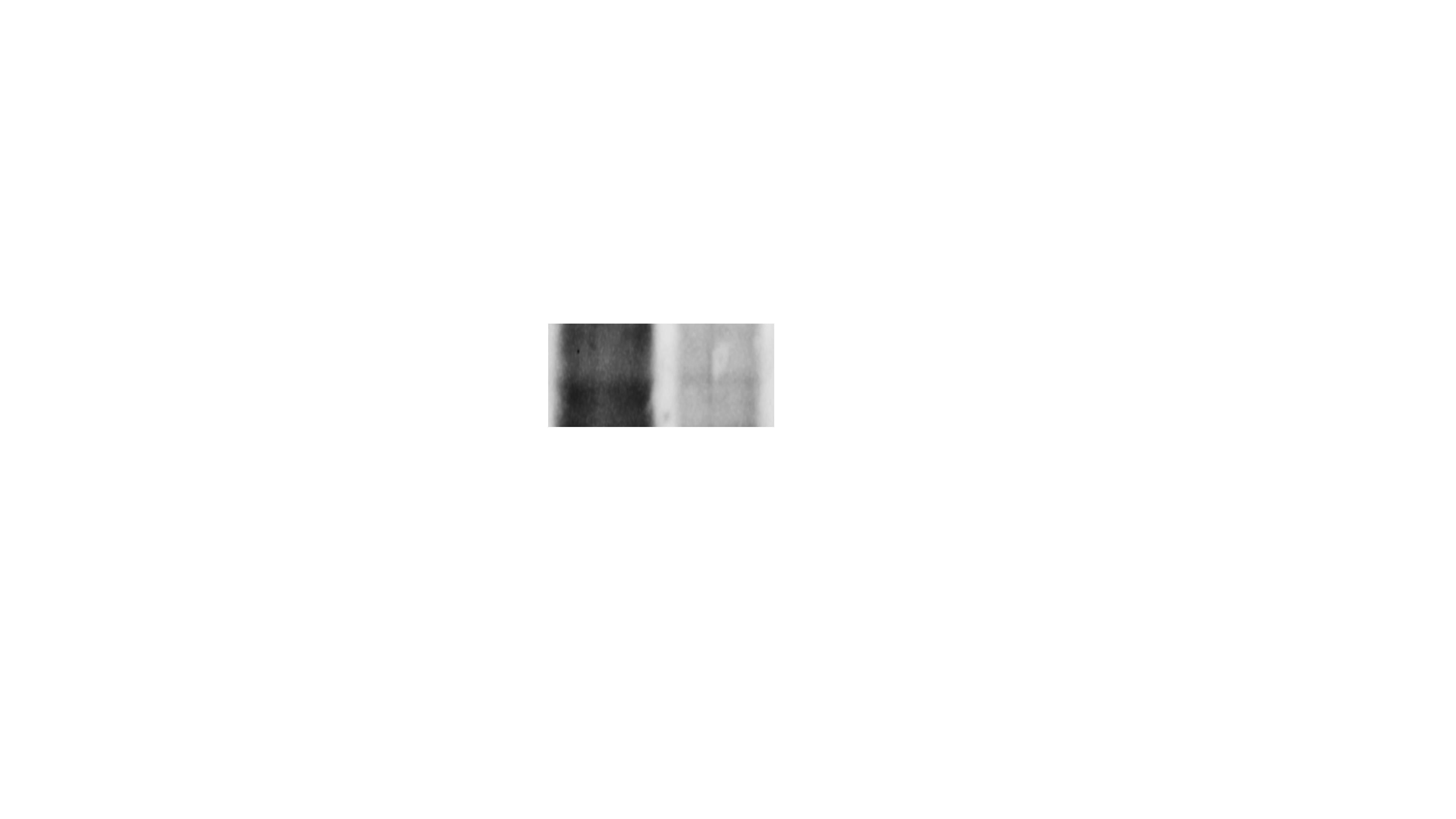

Supplement: Source data 1. [file elife-74765-data1.zip › 100355_1_supp_data_2358340_r6ybjl/Figure 6 - source data 2.TIF]

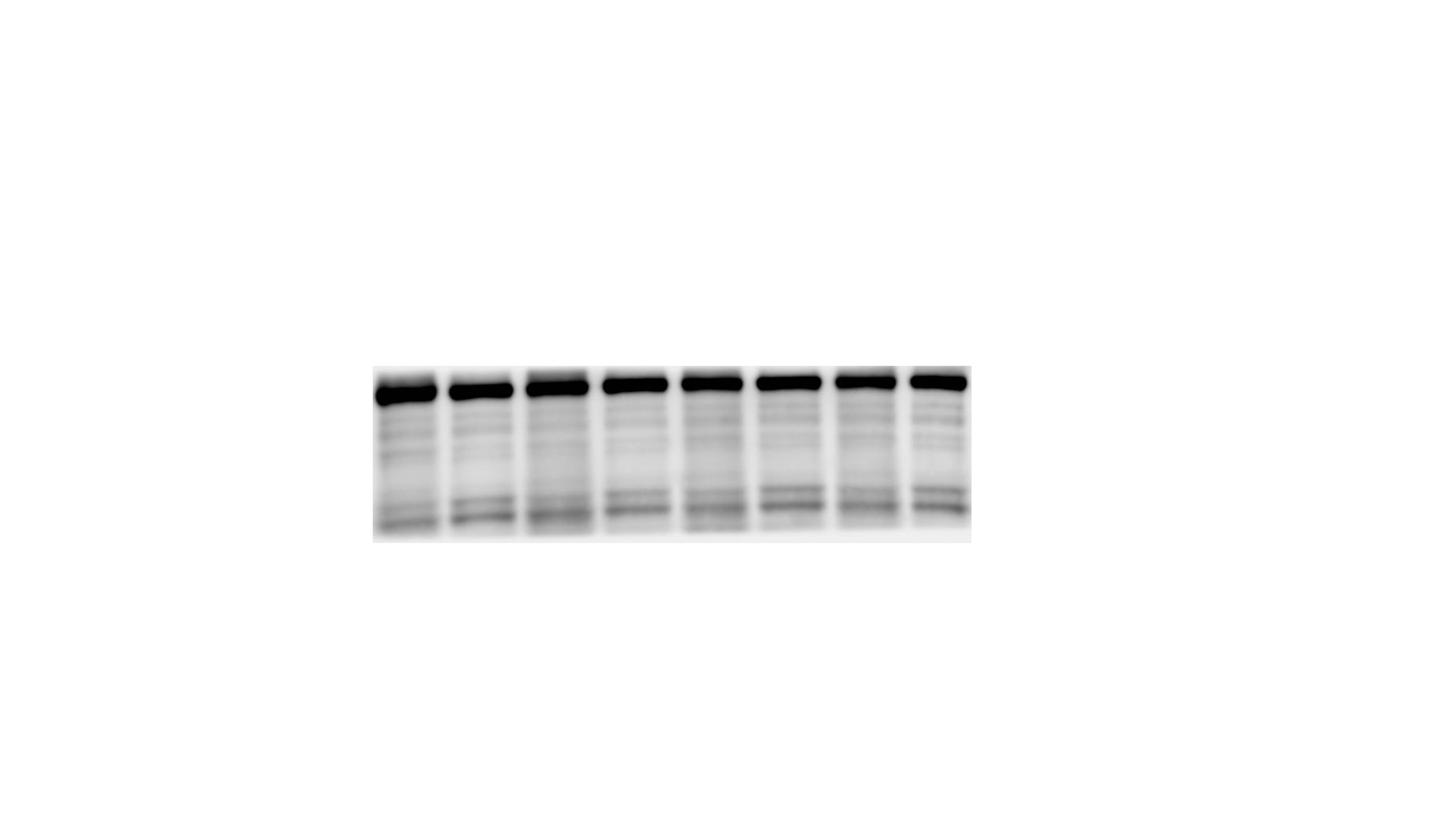

Supplement: Source data 1. [file elife-74765-data1.zip › 100355_1_supp_data_2358340_r6ybjl/Figure 1 - source data 7.TIF]

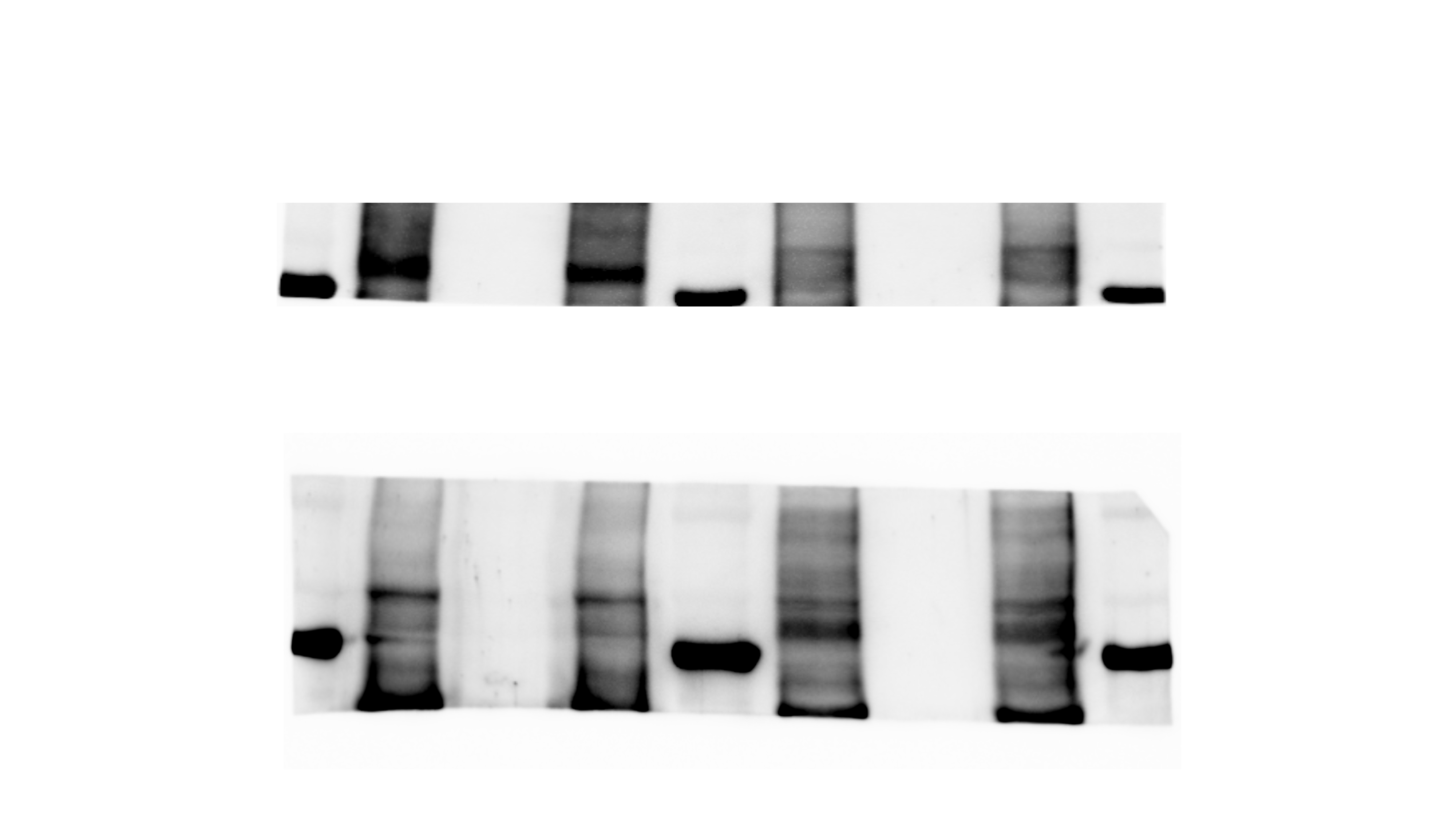

Supplement: Source data 1. [file elife-74765-data1.zip › 100355_1_supp_data_2358340_r6ybjl/Figure 4 - source data 1.tif]

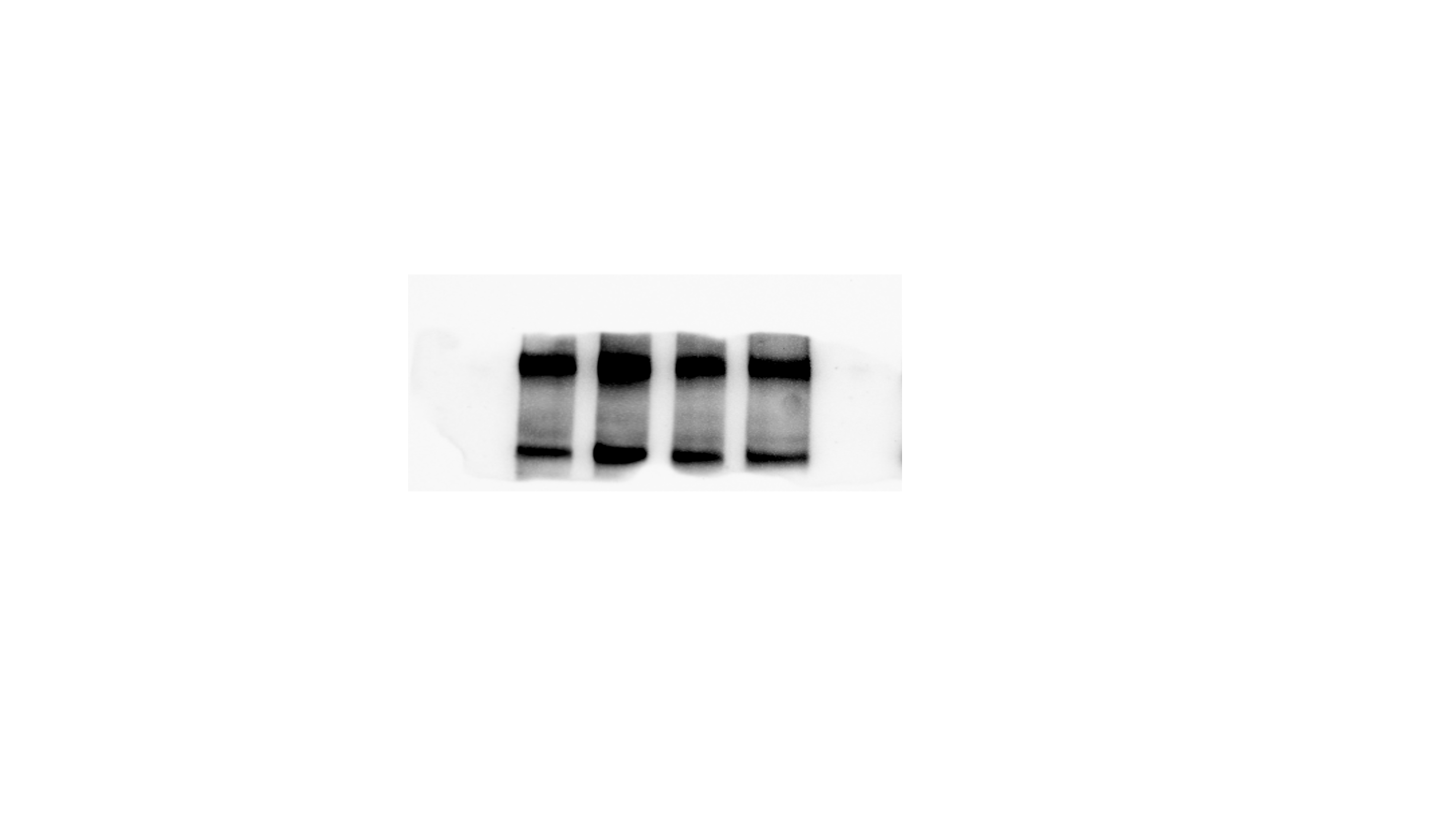

Supplement: Source data 1. [file elife-74765-data1.zip › 100355_1_supp_data_2358340_r6ybjl/Figure 2 - source data 1.TIF]

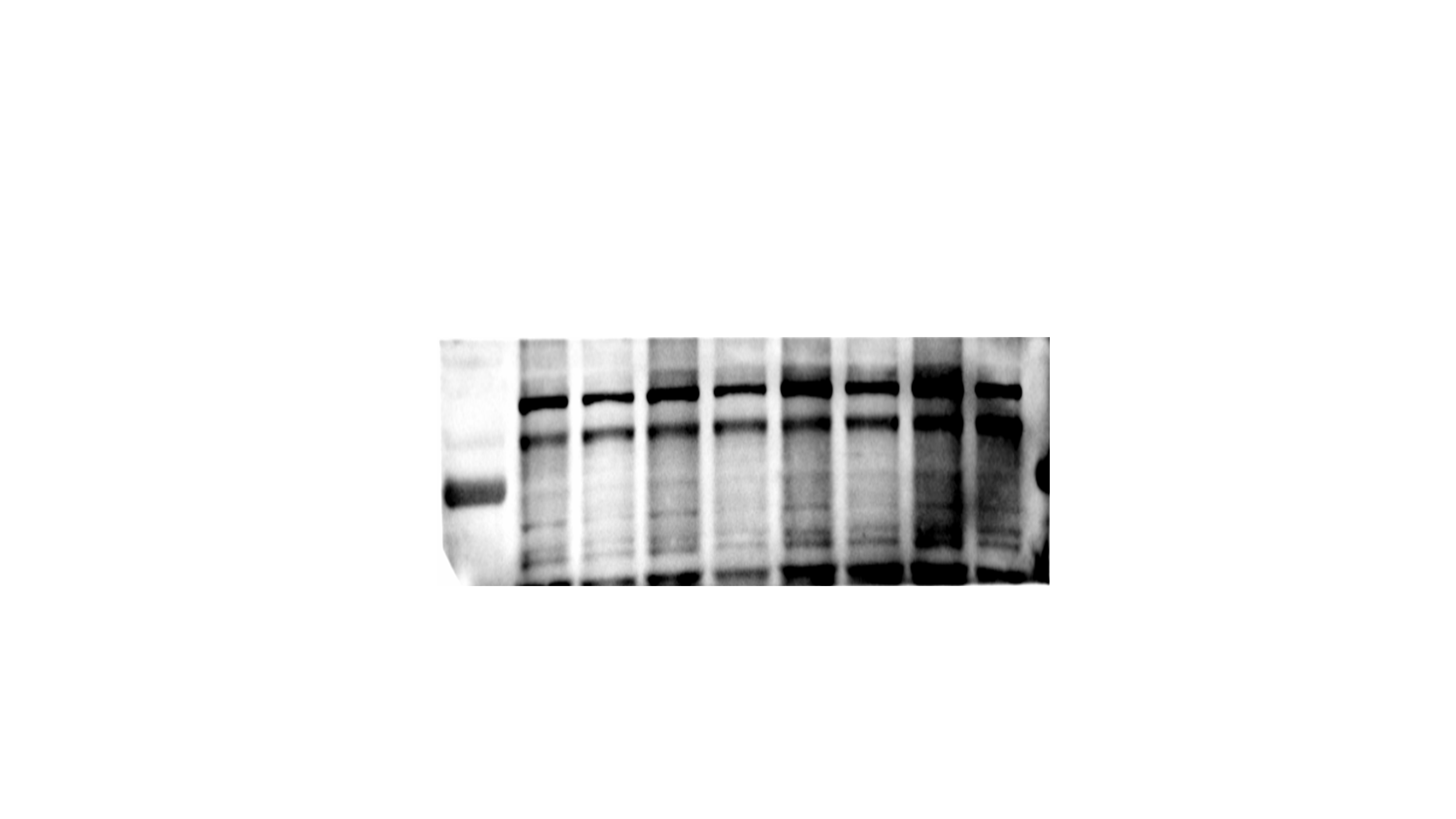

Supplement: Source data 1. [file elife-74765-data1.zip › 100355_1_supp_data_2358340_r6ybjl/Figure 1 - source data 3.TIF]

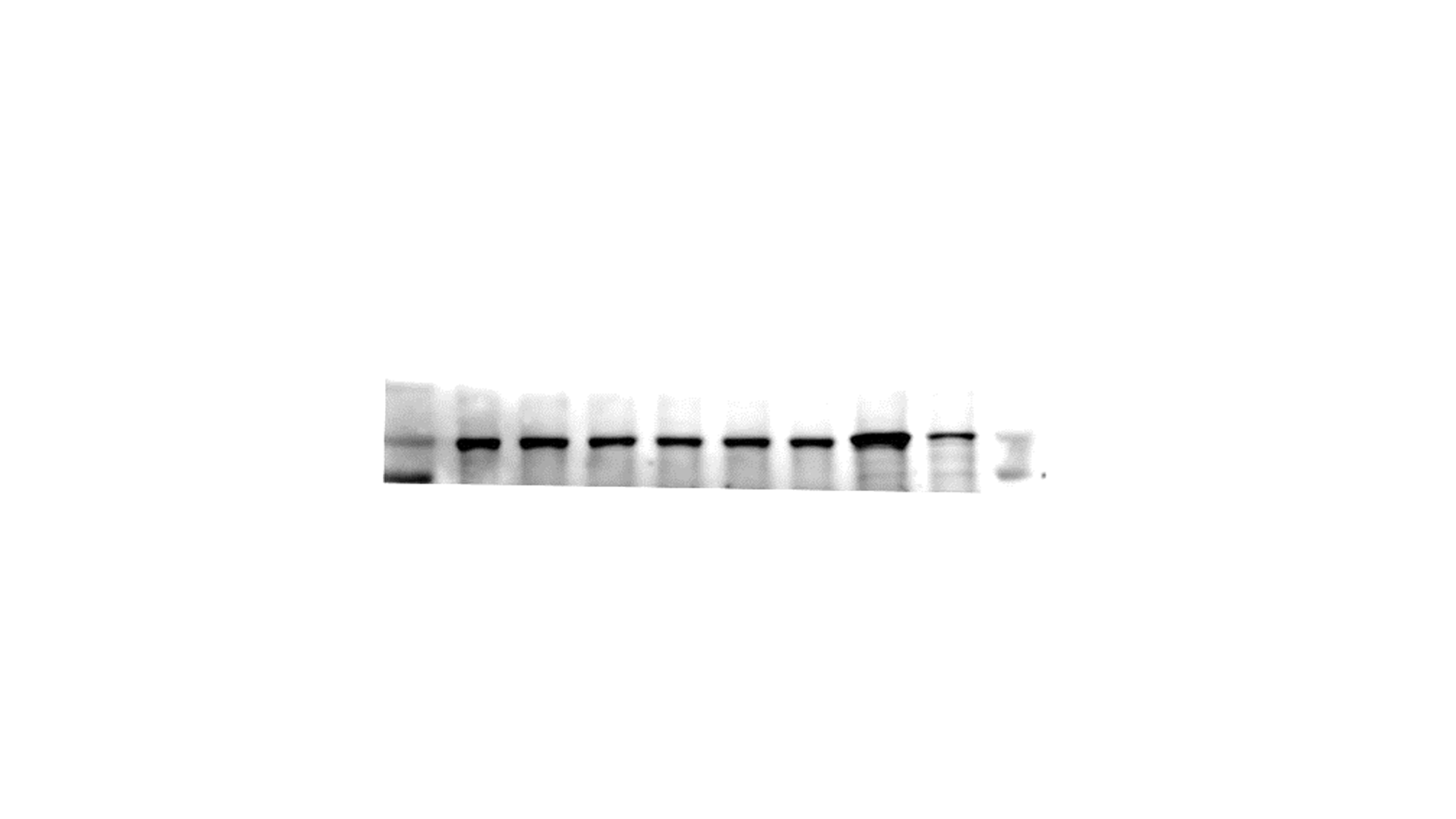

Supplement: Source data 1. [file elife-74765-data1.zip › 100355_1_supp_data_2358340_r6ybjl/Figure 1 - source data 2.TIF]

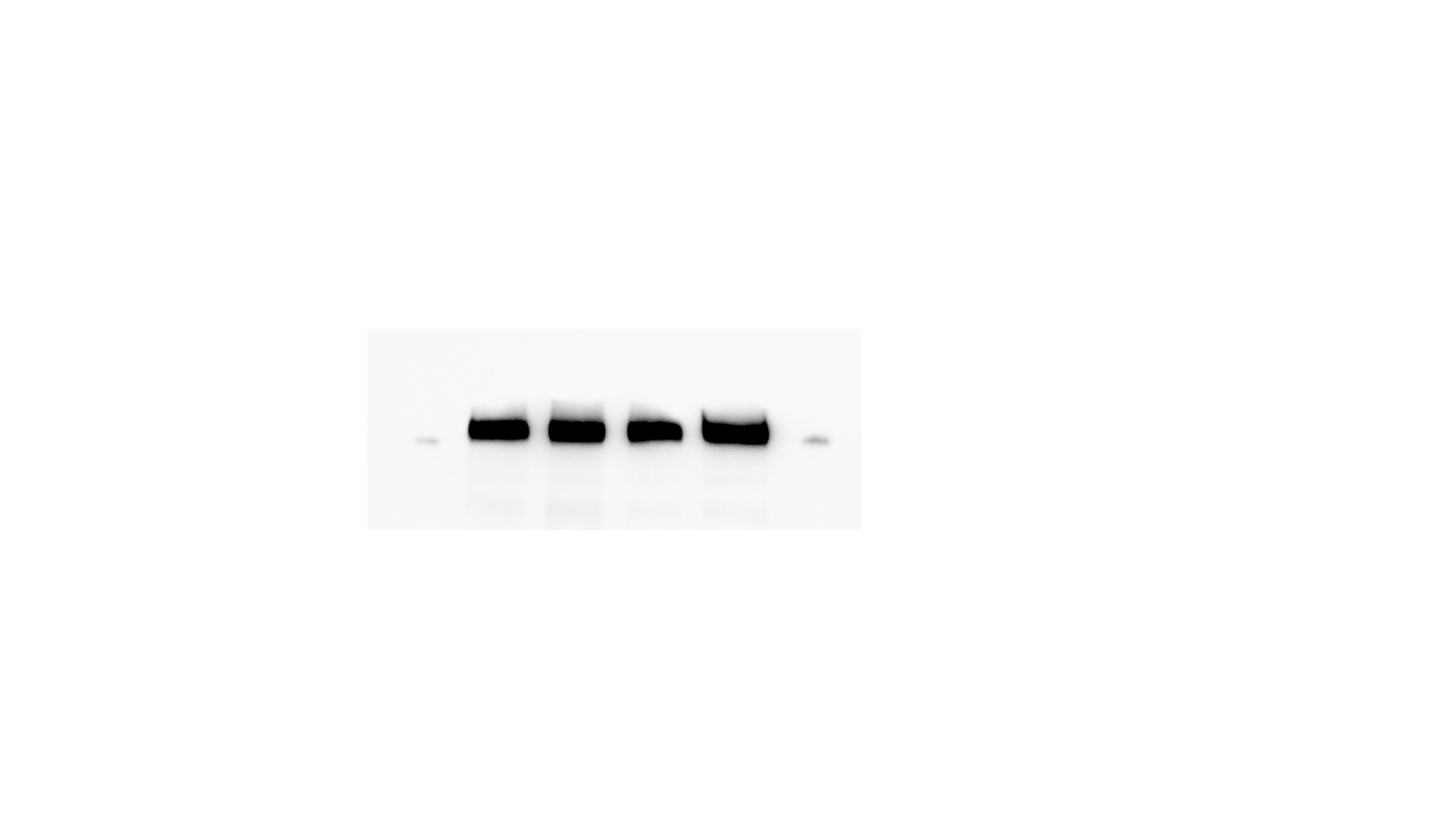

Supplement: Source data 1. [file elife-74765-data1.zip › 100355_1_supp_data_2358340_r6ybjl/Figure 2 - source data 2.TIF]

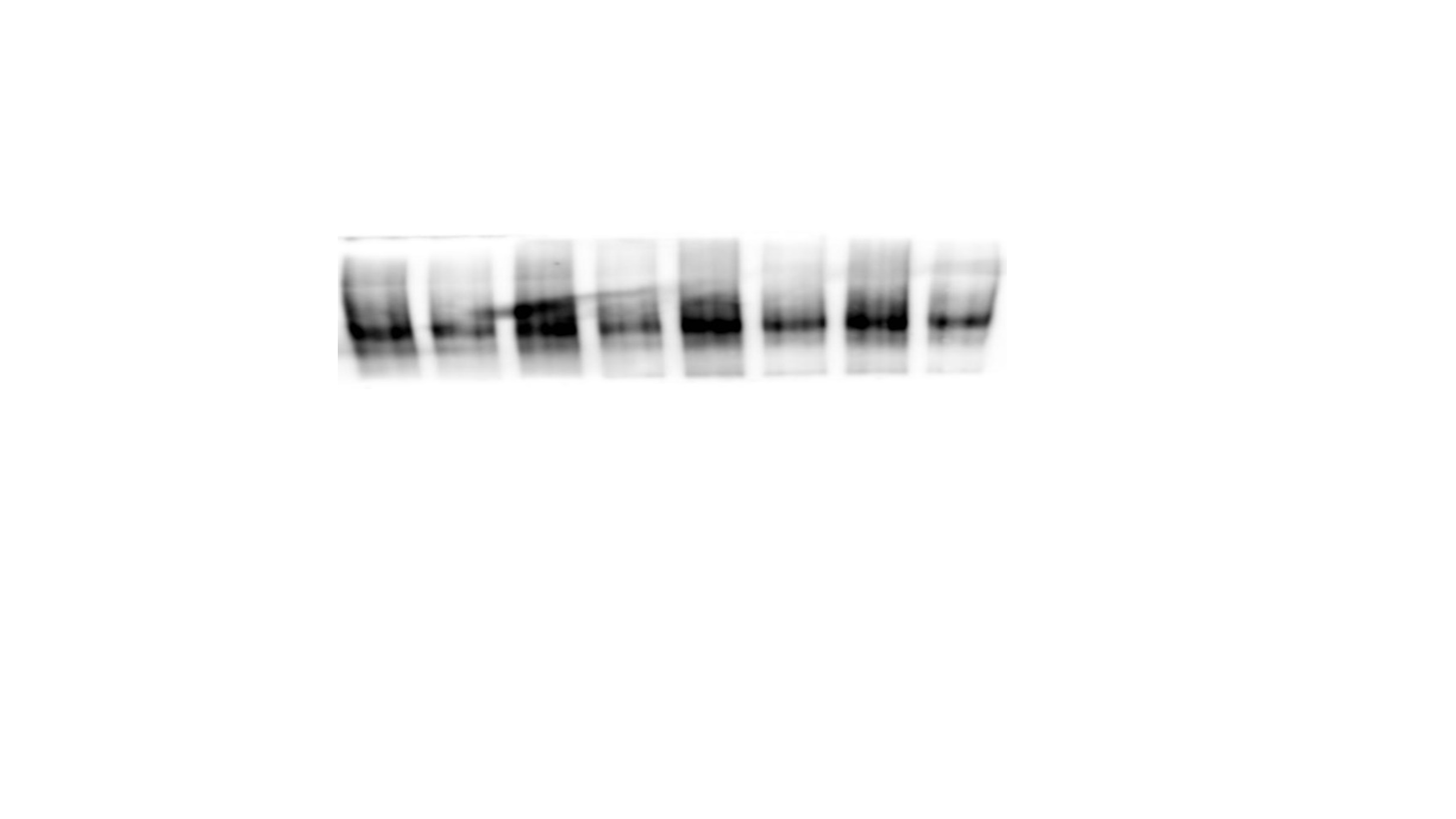

Supplement: Source data 1. [file elife-74765-data1.zip › 100355_1_supp_data_2358340_r6ybjl/Figure 1 - source data 1.TIF]

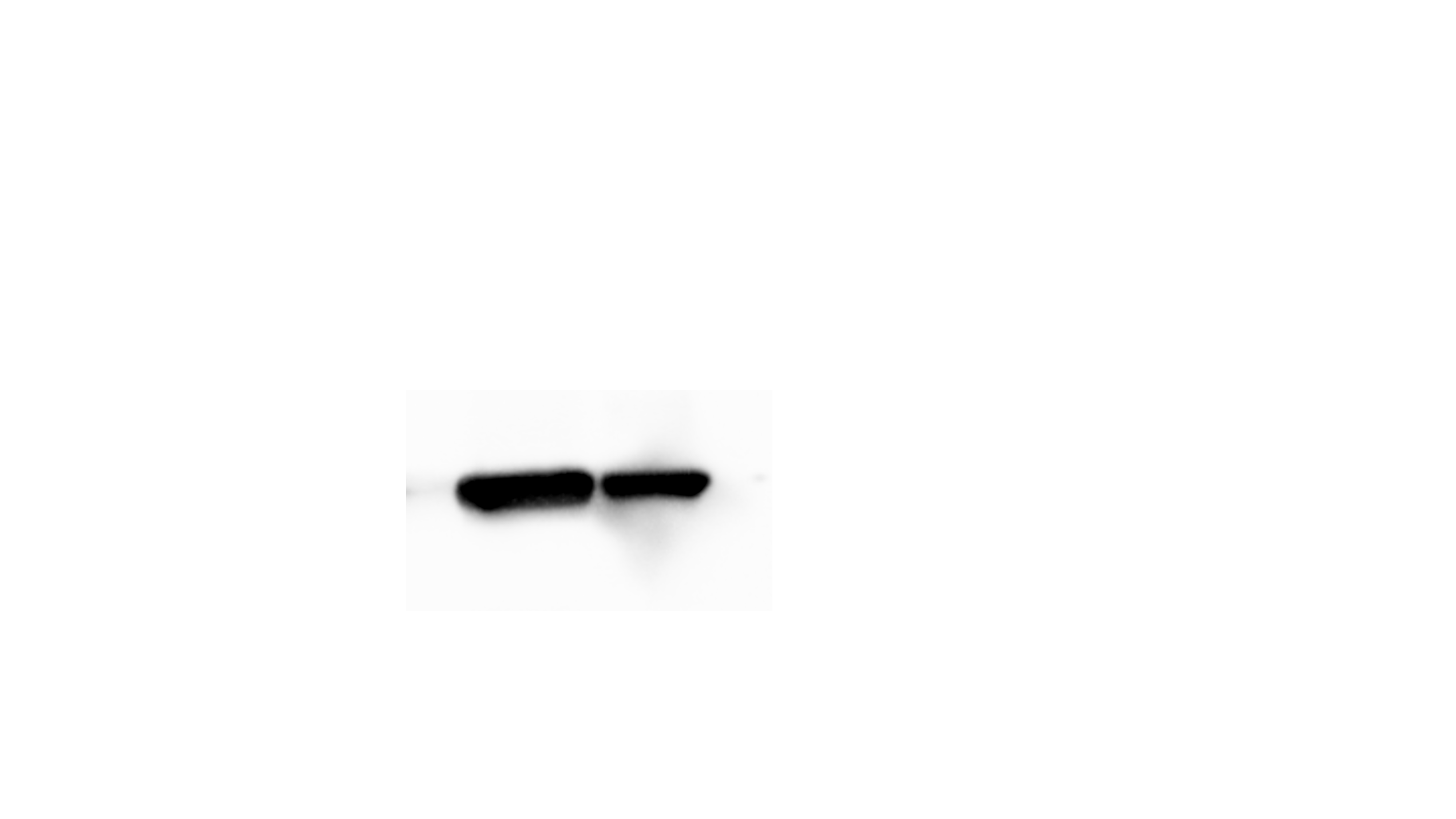

Supplement: Source data 1. [file elife-74765-data1.zip › 100355_1_supp_data_2358340_r6ybjl/Figure 6 - source data 4.TIF]

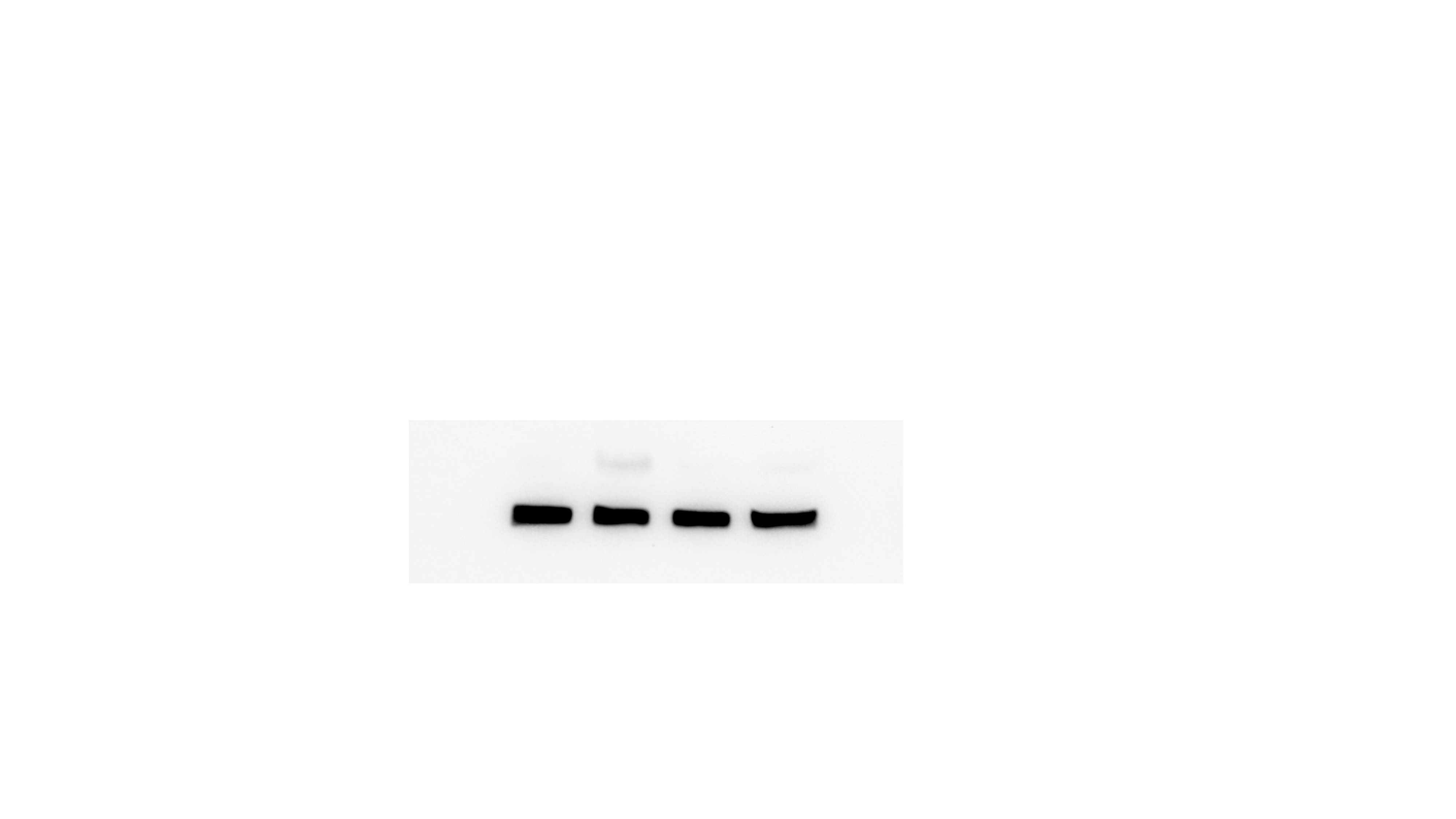

Supplement: Source data 1. [file elife-74765-data1.zip › 100355_1_supp_data_2358340_r6ybjl/Figure 2 - source data 3.TIF]

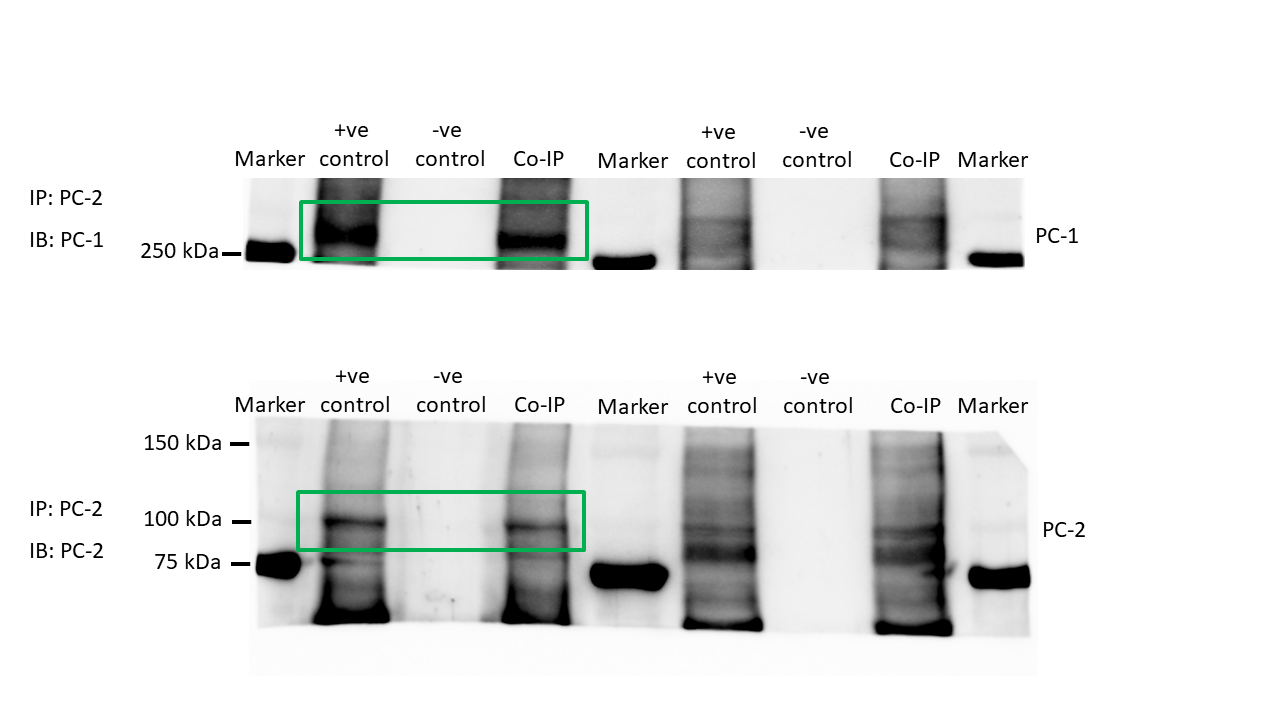

Supplement: Source data 2. [file elife-74765-data2.zip › Figure 4-source data 1.tif]

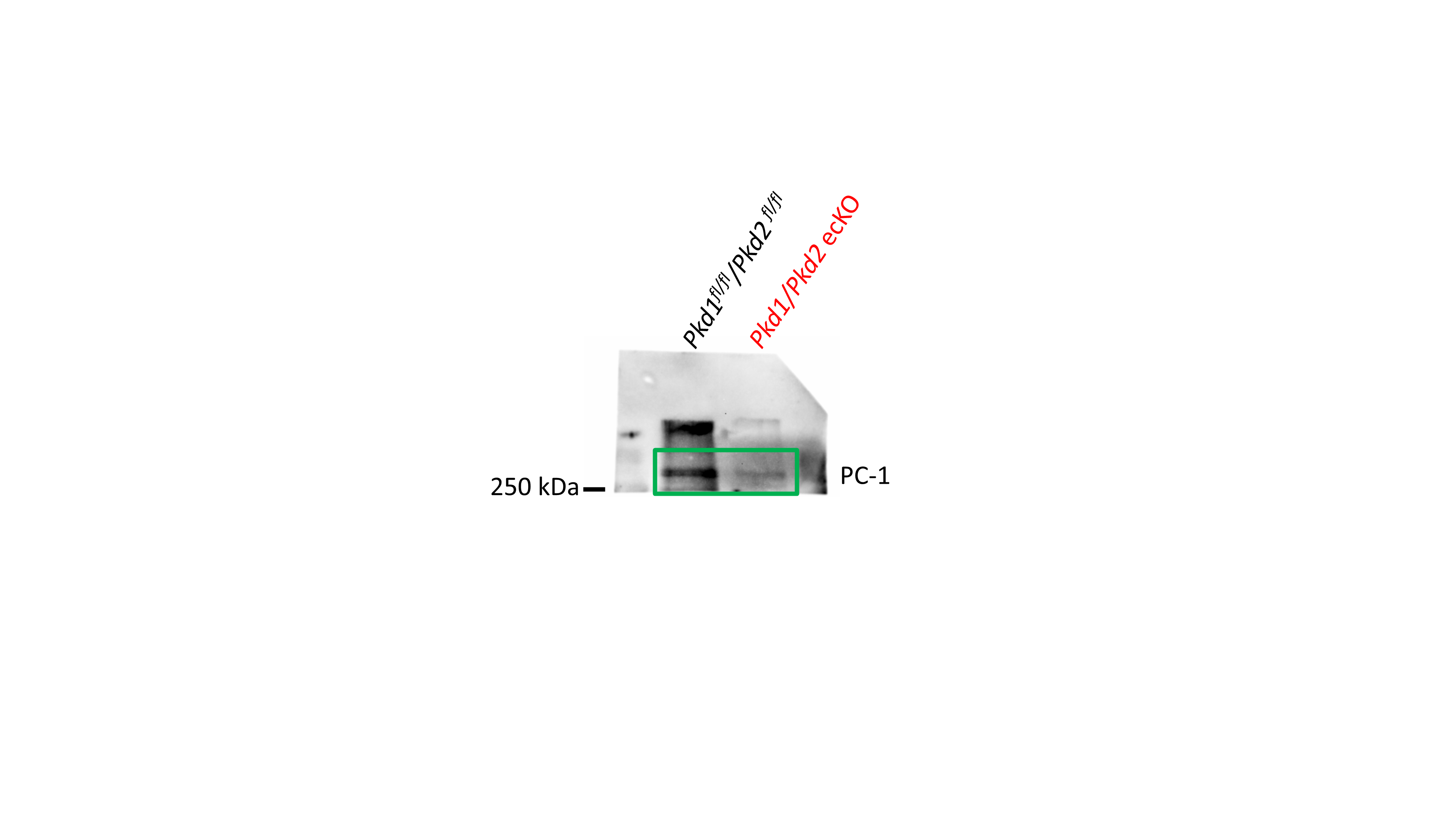

Supplement: Source data 2. [file elife-74765-data2.zip › Figure 6-source data 1.tif]

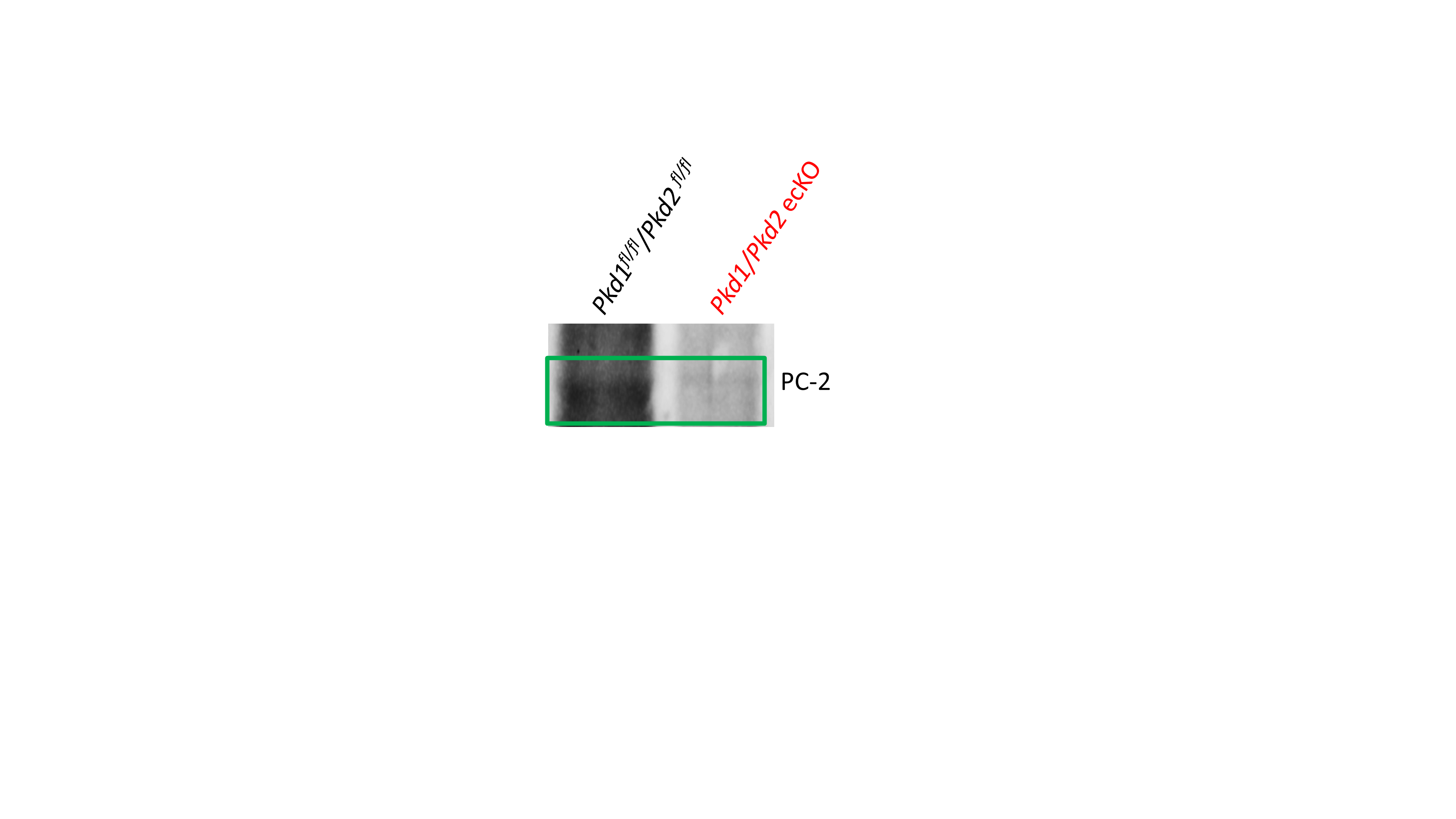

Supplement: Source data 2. [file elife-74765-data2.zip › Figure 6-source data 2.TIF]

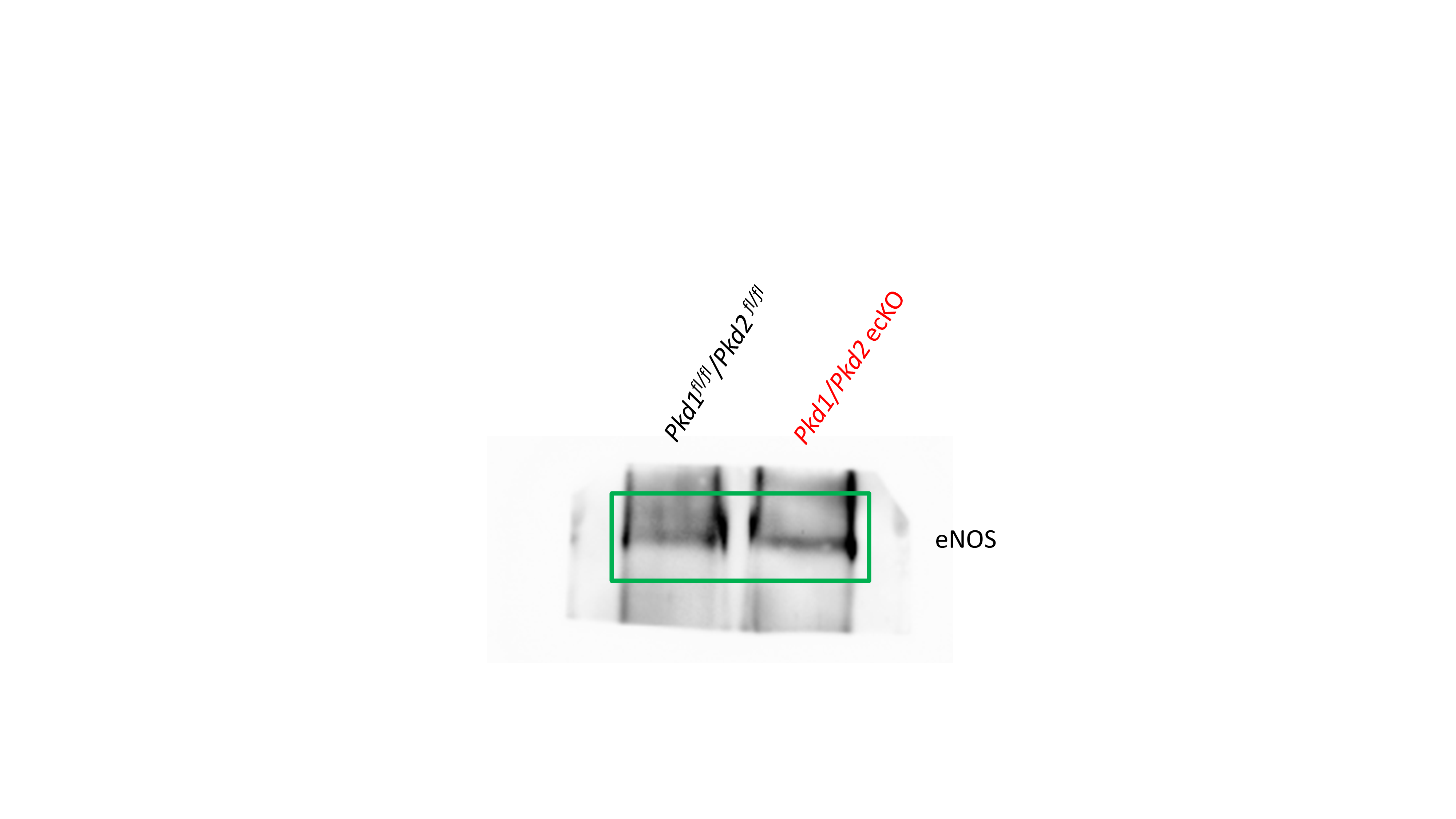

Supplement: Source data 2. [file elife-74765-data2.zip › Figure 6-source data 3.TIF]

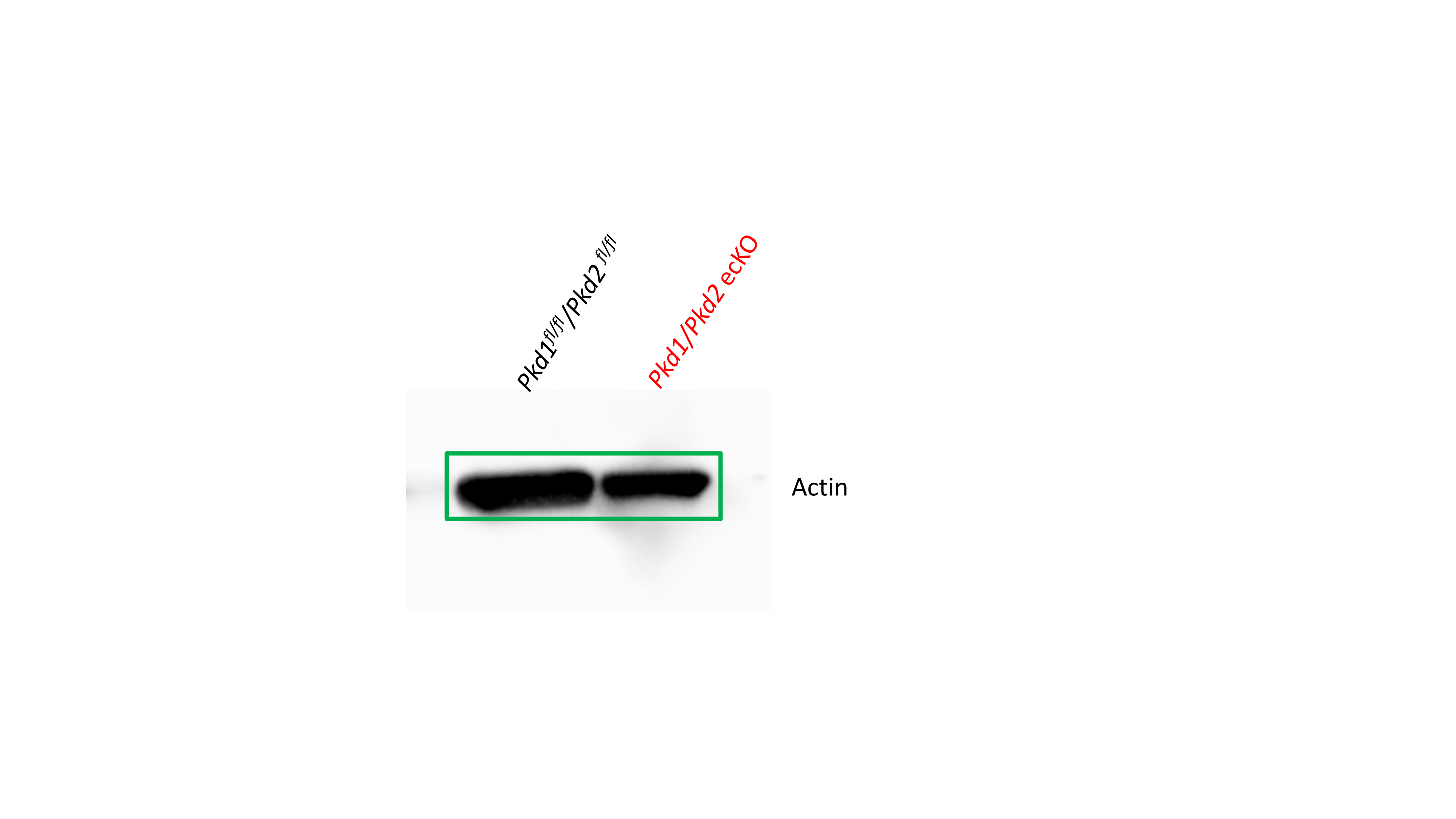

Supplement: Source data 2. [file elife-74765-data2.zip › Figure 6-source data 4.TIF]

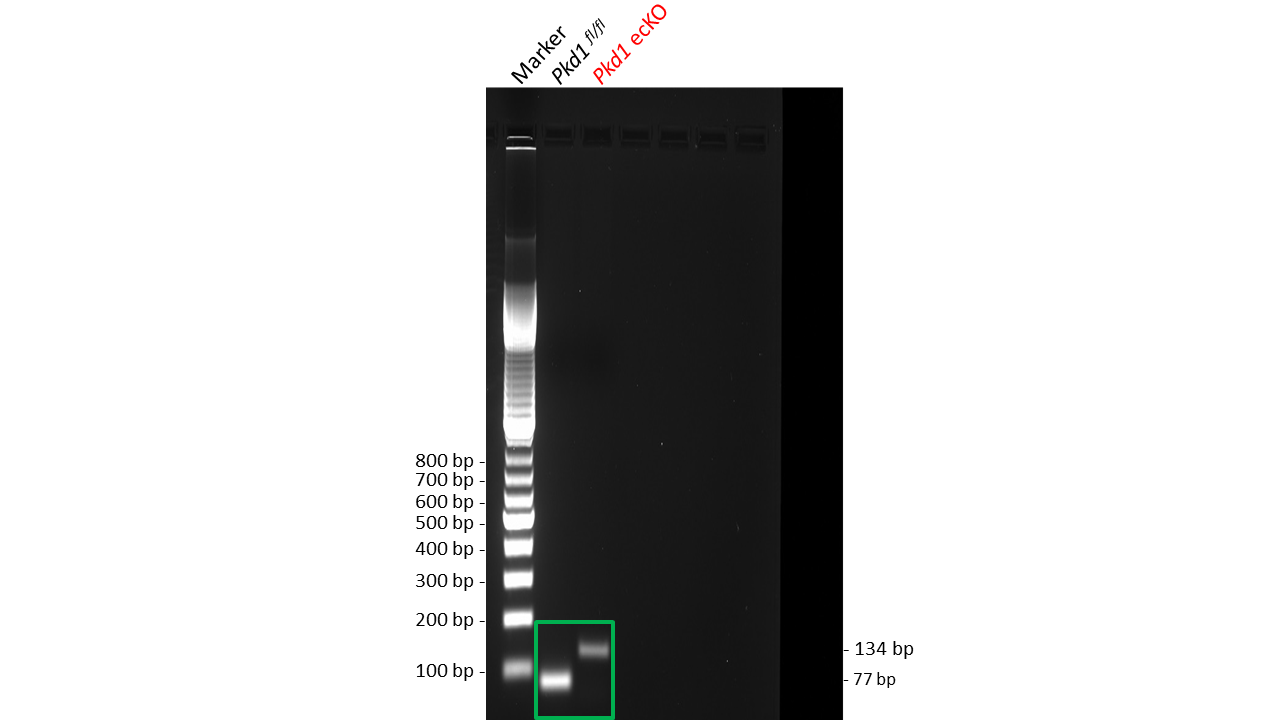

Supplement: Source data 2. [file elife-74765-data2.zip › Figure 1-Figure Supplement 1-source data 1.tif]

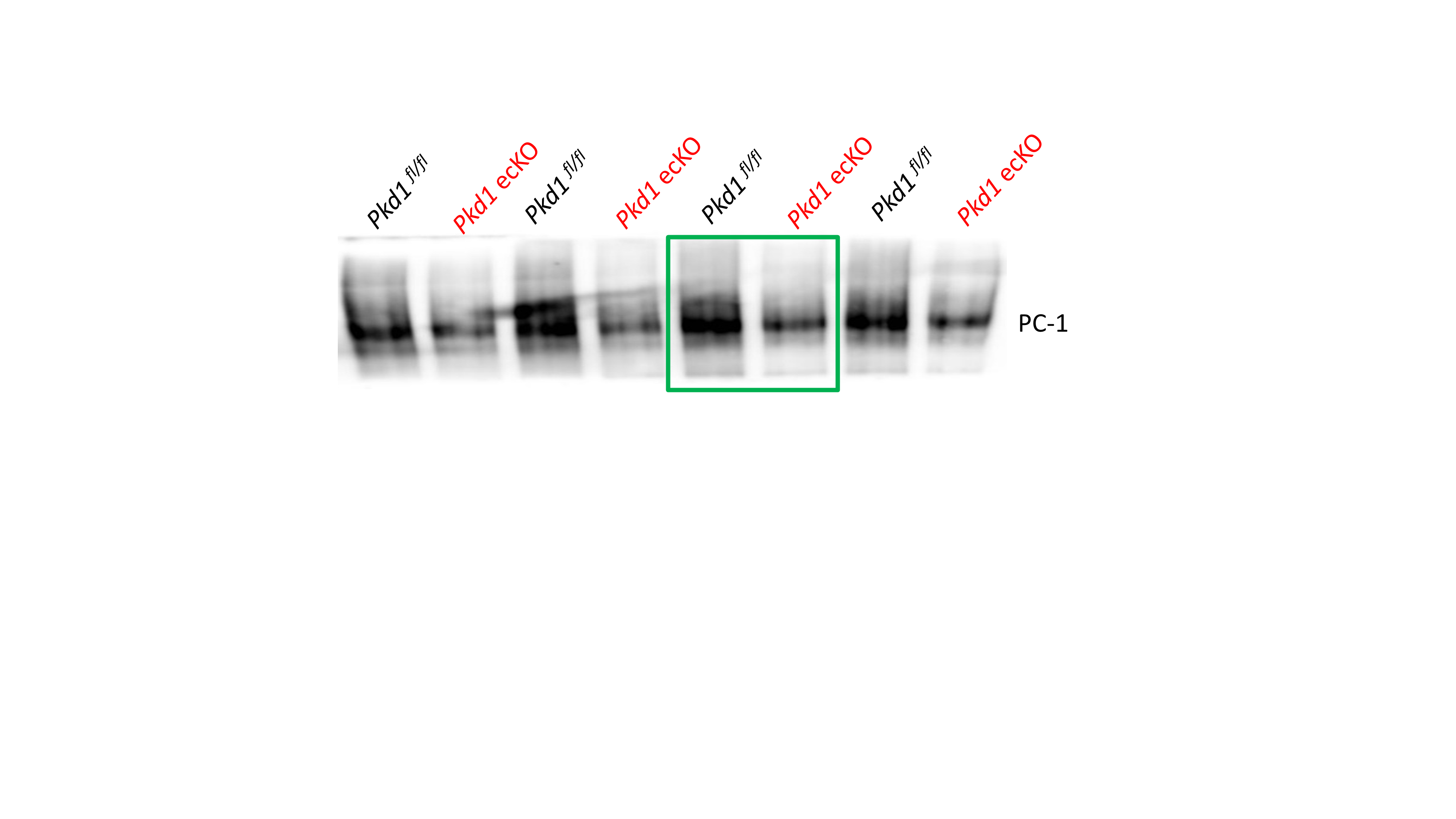

Supplement: Source data 2. [file elife-74765-data2.zip › figure 1-source data 1.TIF]

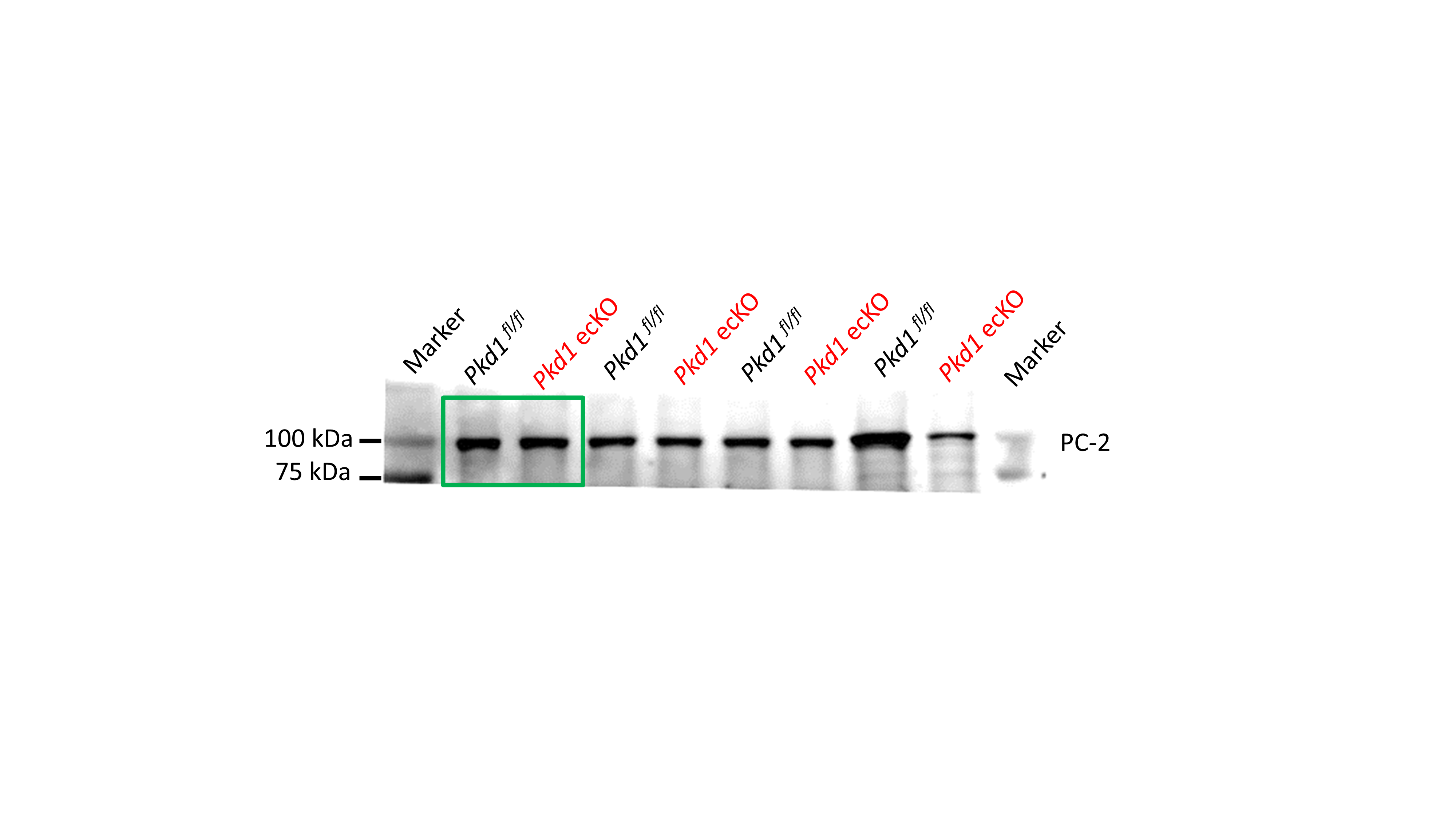

Supplement: Source data 2. [file elife-74765-data2.zip › Figure 1-source data 2.TIF]

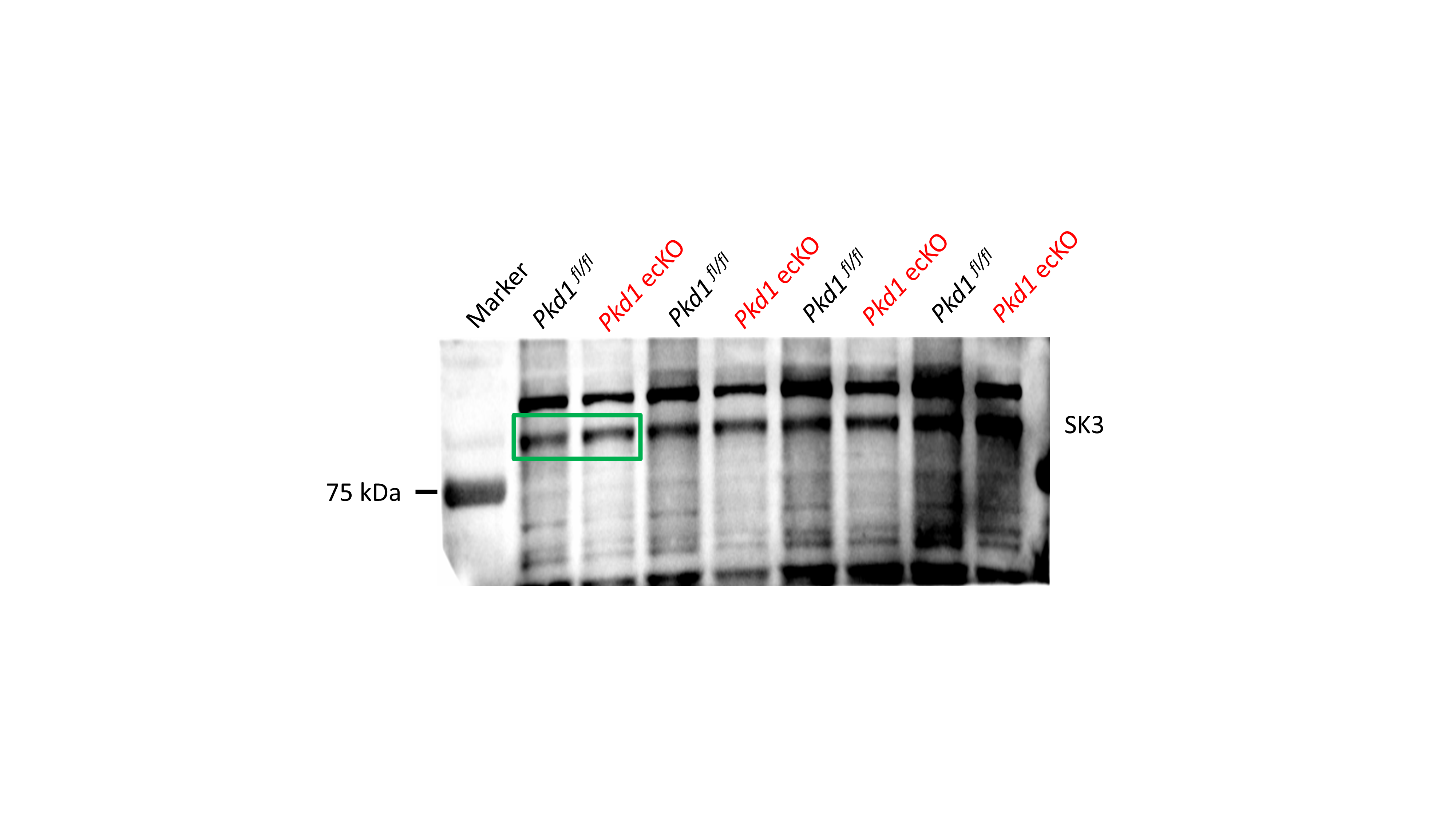

Supplement: Source data 2. [file elife-74765-data2.zip › Figure 1-source data 3.tif]

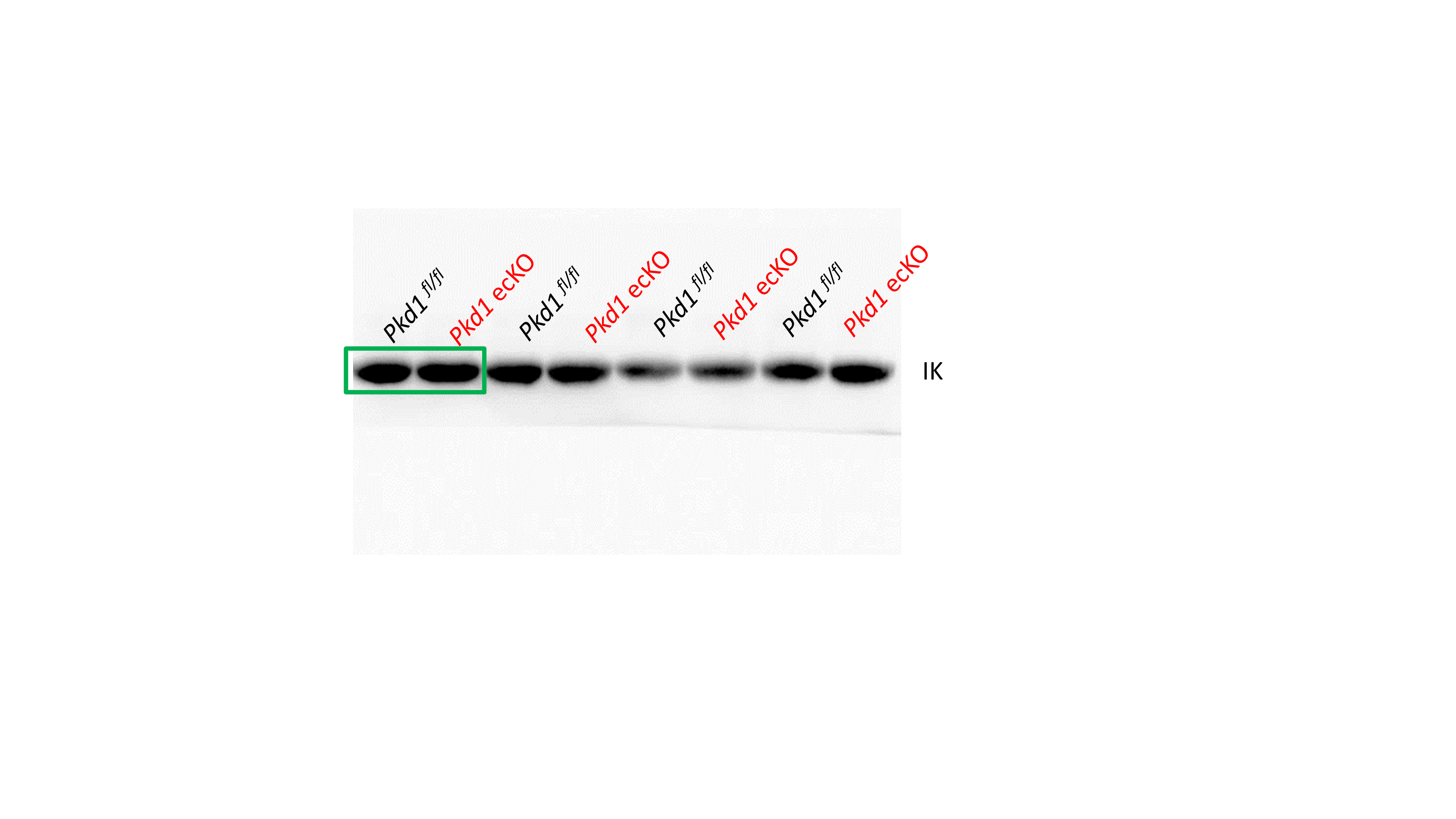

Supplement: Source data 2. [file elife-74765-data2.zip › Figure 1-source data 4.TIF]

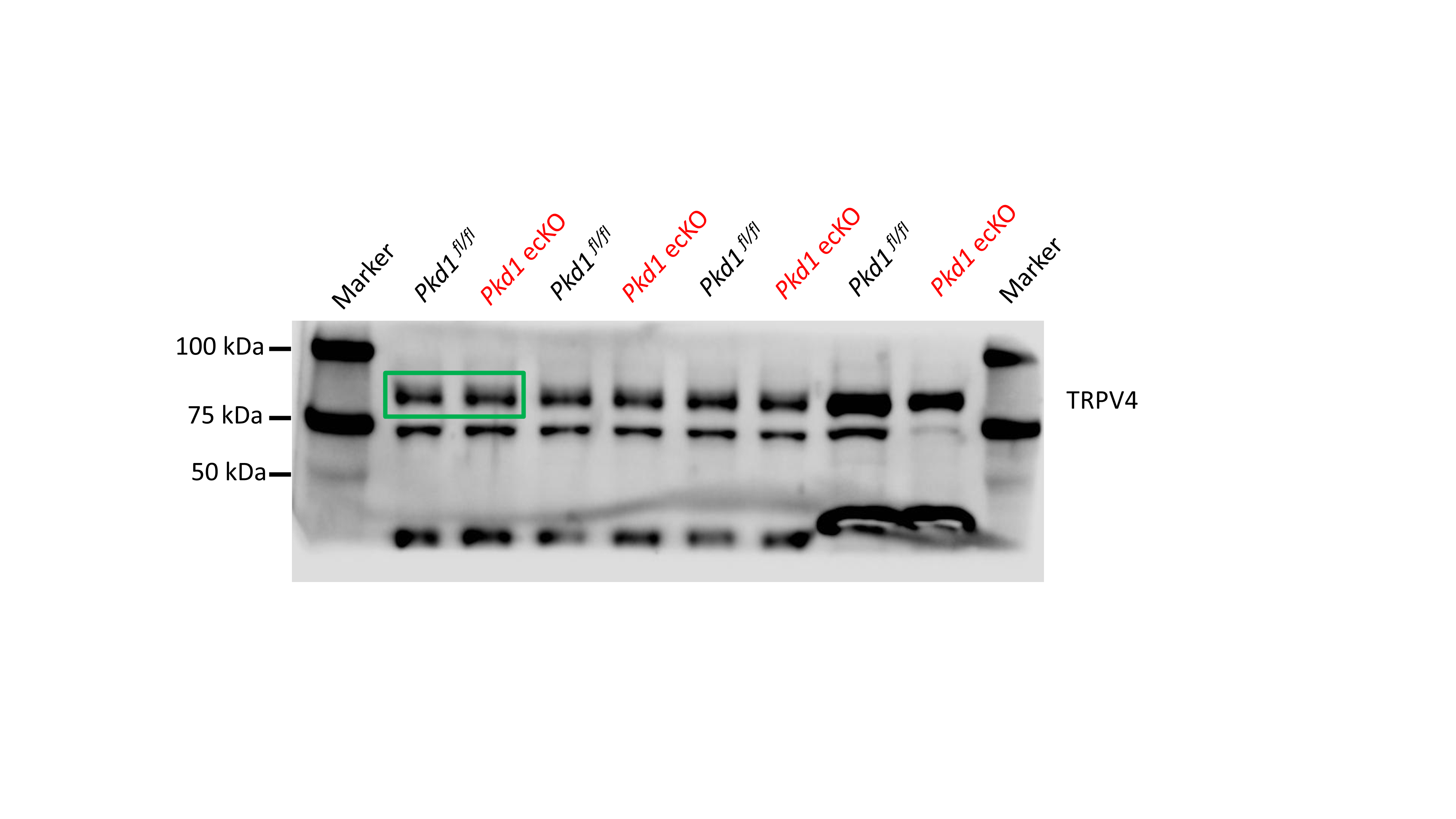

Supplement: Source data 2. [file elife-74765-data2.zip › figure 1-source data 5.TIF]

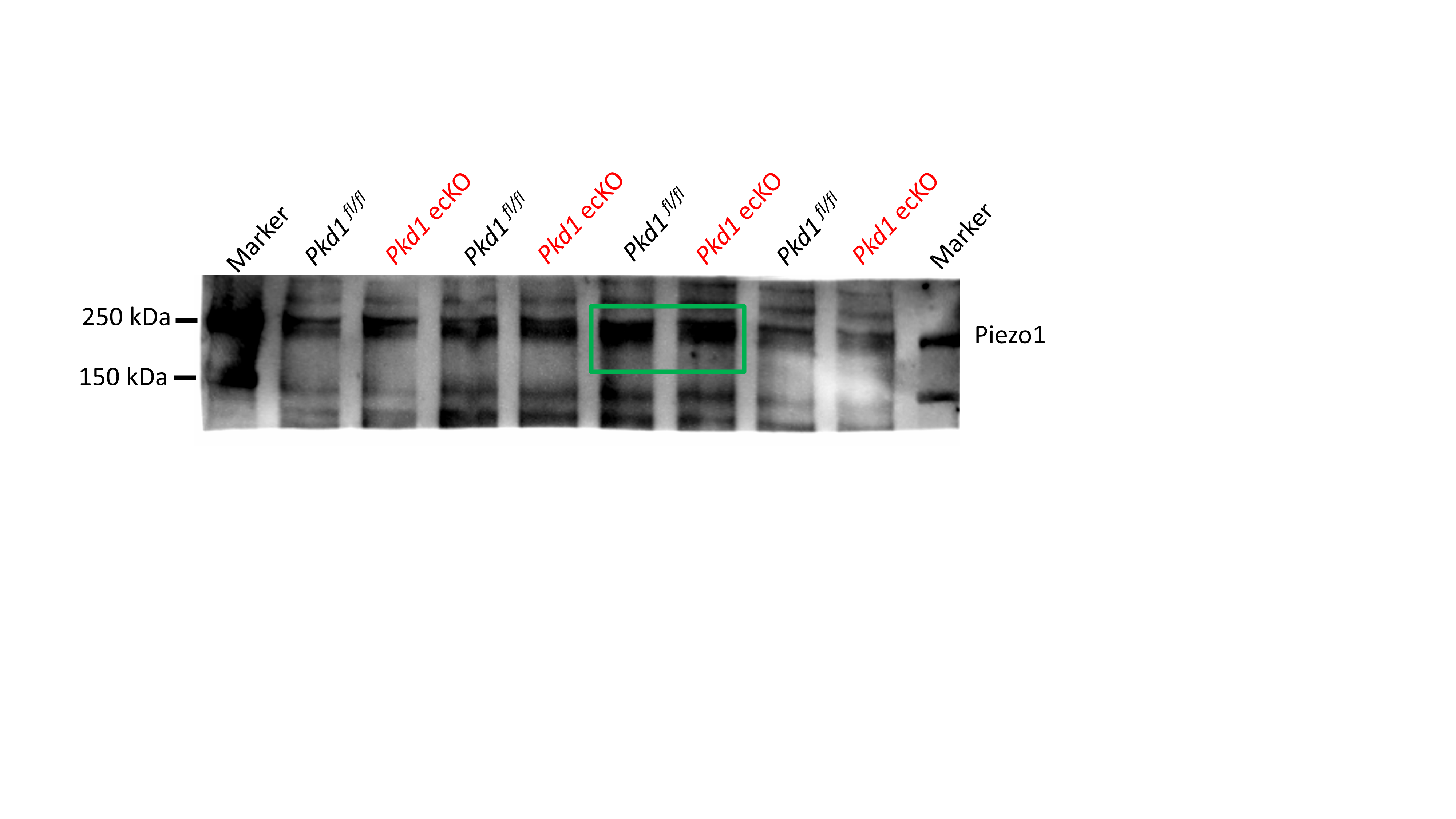

Supplement: Source data 2. [file elife-74765-data2.zip › figure 1-source data 6.TIF]

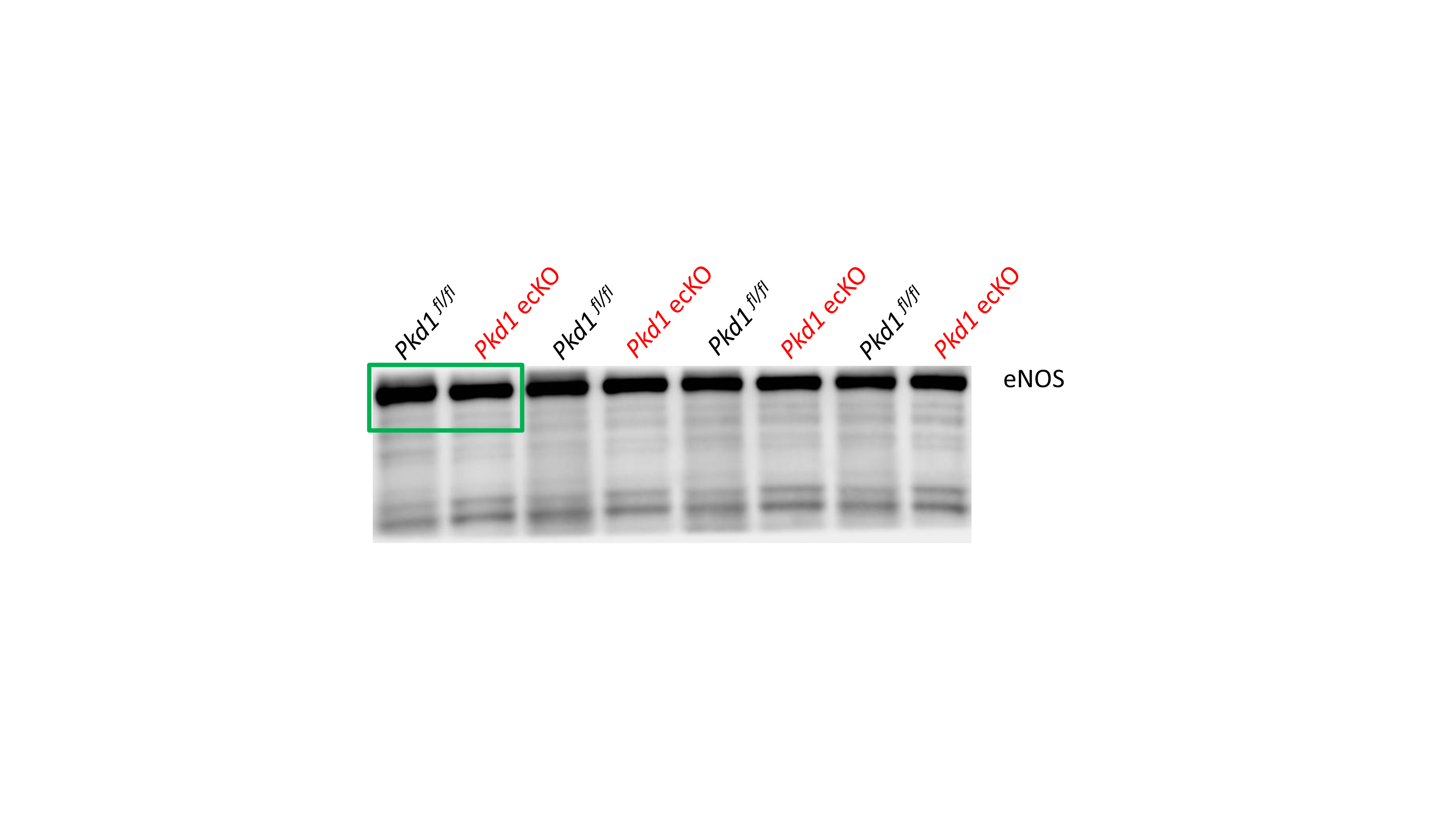

Supplement: Source data 2. [file elife-74765-data2.zip › Figure 1-source data 7.TIF]

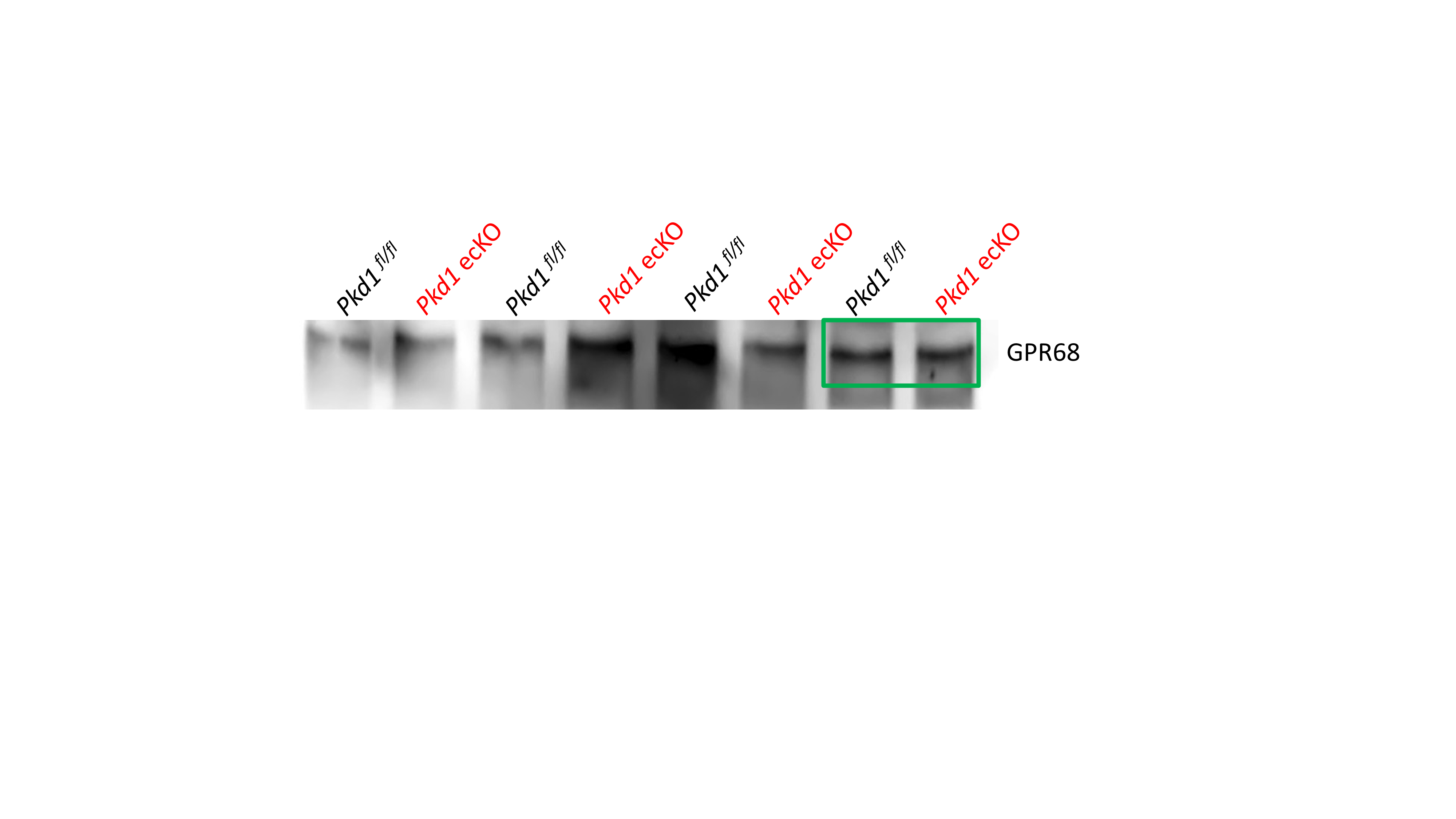

Supplement: Source data 2. [file elife-74765-data2.zip › figure 1-source data 8.TIF]

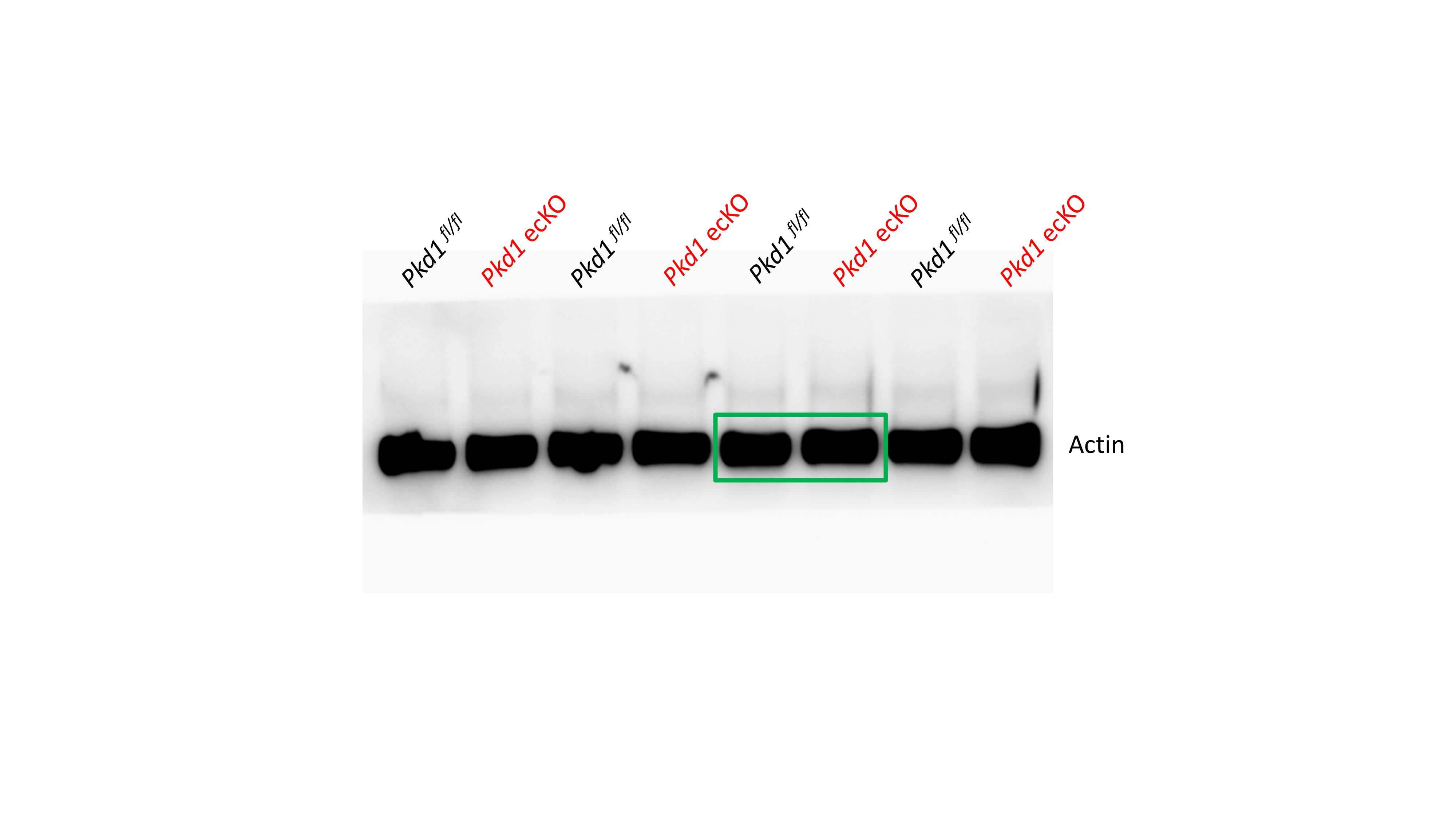

Supplement: Source data 2. [file elife-74765-data2.zip › Figure 1-source data 9.TIF]

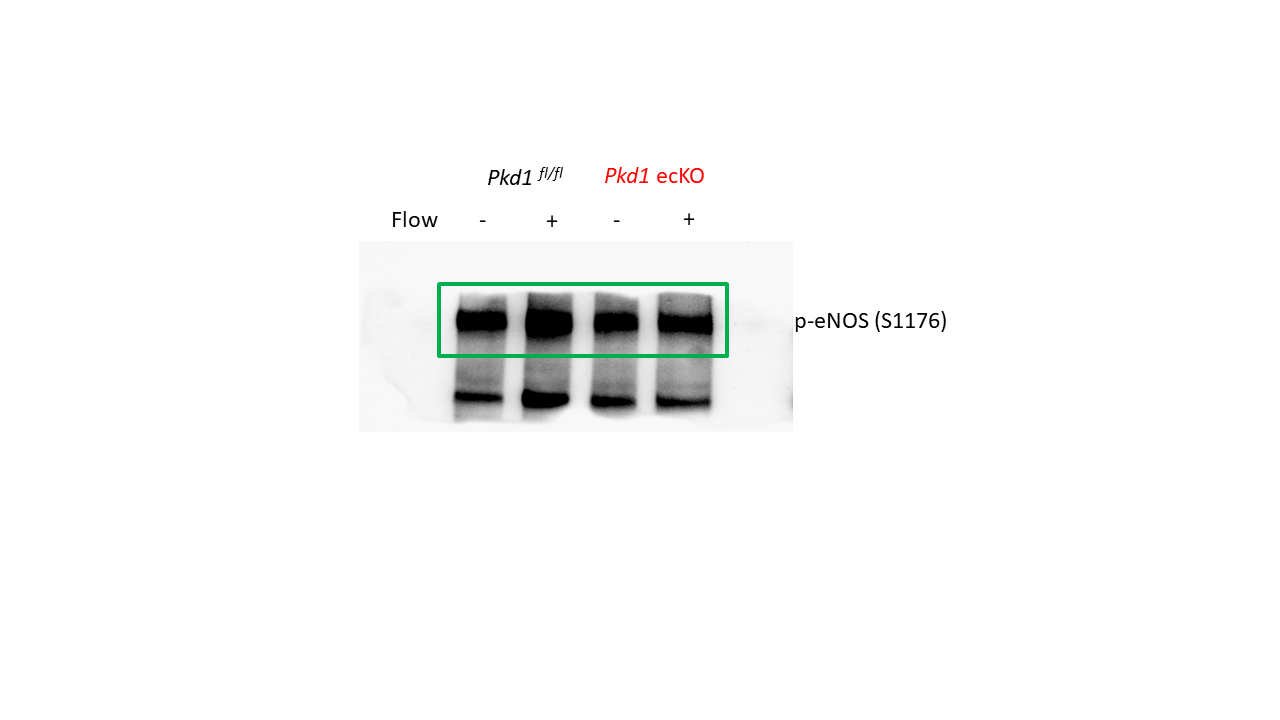

Supplement: Source data 2. [file elife-74765-data2.zip › Figure 2-source data 1.tif]

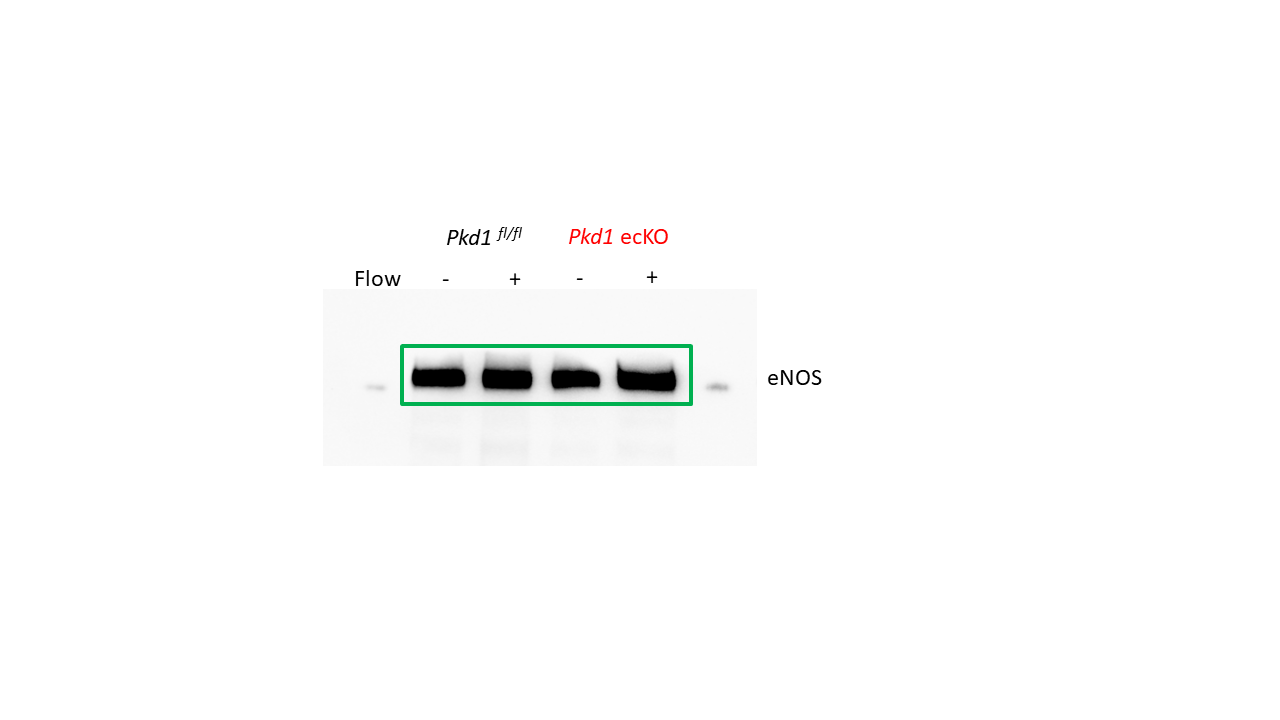

Supplement: Source data 2. [file elife-74765-data2.zip › Figure 2-source data 2.tif]

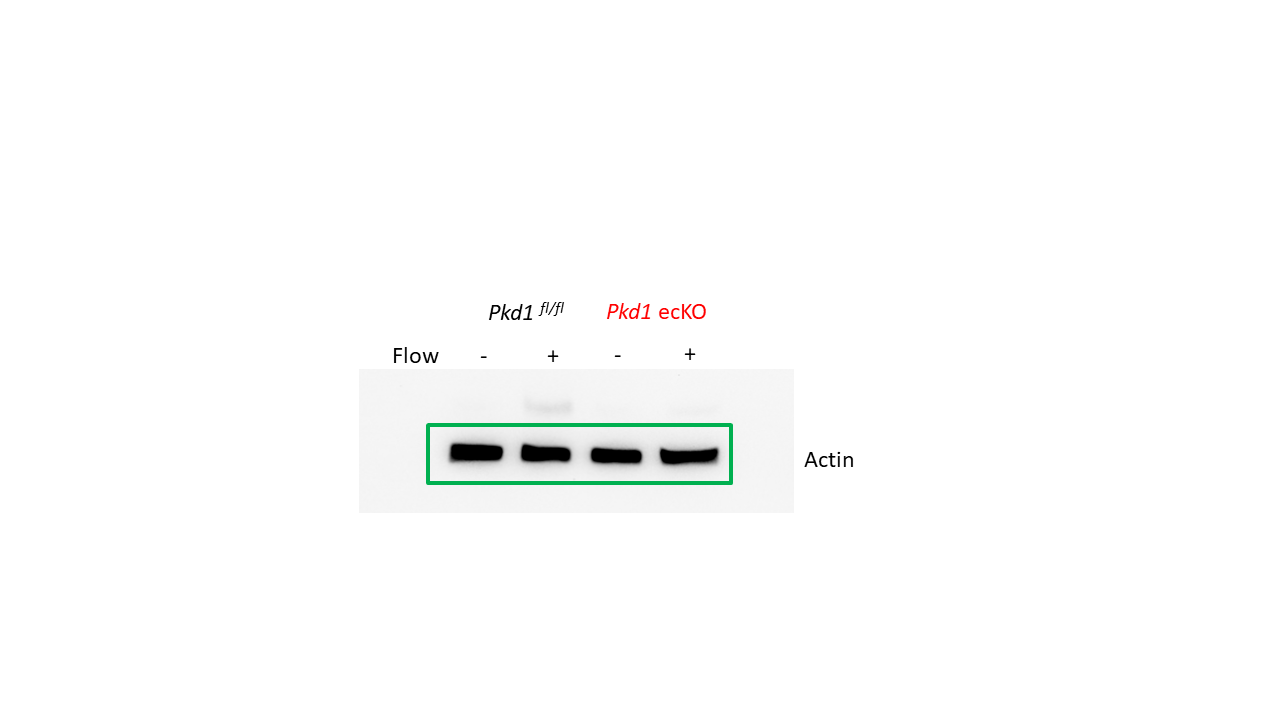

Supplement: Source data 2. [file elife-74765-data2.zip › Figure 2-source data 3.tif]
